# Integrative methylome and transcriptome analysis reveals genotype and sequence context-specific responses to aluminum stress in rice

**DOI:** 10.3389/fpls.2026.1896320

**Published:** 2026-07-31

**Authors:** Jenny Johana Gallo-Franco, Chrystian Camilo Sosa, Frank Johannes, Thaura Ghneim-Herrera, Mauricio Quimbaya

**Affiliations:** 1 International Center for Tropical Agriculture (CIAT), Cali, Colombia; 2 Department of Natural Sciences and Mathematics, Pontificia Universidad Javeriana-Cali, Cali, Colombia; 3 Evolution, Ecology and Conservation Research Group EECO, Biology Program, Faculty of Basic Sciences, Armenia, Colombia; 4 School of Life Sciences, Technical University of Munich, Freising, Germany; 5 Department of Biological Sciences, Universidad ICESI, Cali, Colombia

**Keywords:** abiotic stress, aluminum tolerance, bisulfite sequencing, differential expression, DNA methylation, RNA-seq

## Abstract

**Introduction:**

Aluminum (Al) toxicity in acidic soils is a major constraint to rice production worldwide. However, the epigenetic mechanisms underlying genotypic differences in Al tolerance remain largely unexplored.

**Methods:**

We integrated whole-genome bisulfite sequencing (WGBS) and RNA sequencing (RNA-seq) to characterize DNA methylation dynamics and their relationship with gene expression in two contrasting *Oryza sativa* genotypes, Al-tolerant Azucena and Al-susceptible BGI, exposed to prolonged Al stress (10 days).

**Results:**

Genome-wide analysis revealed pronounced context-specific methylation changes that differed markedly between genotypes. CHG methylation was broadly reduced under stress in both genotypes, with a stronger response in BGI, whereas CHH methylation showed a genome-wide increase in BGI but only modest changes in Azucena, indicating more extensive epigenomic perturbation in the susceptible background. At the local level, differentially methylated regions (DMRs) were predominantly hypomethylated across all cytosine contexts and, particularly within the CHG and CHH contexts, were significantly enriched within transposable elements and upstream regulatory regions relative to genomic background, suggesting that stress-induced relaxation of TE silencing and regulatory reprogramming of promoter regions are conserved features of the Al epigenetic response. Integration of DMR and differential expression data identified 71 and 93 genes with both methylation and transcriptional changes in Azucena and BGI, respectively, with only three genes shared between genotypes, all showing opposite transcriptional responses, underscoring the near-complete genotype specificity of the methylation–expression interface. In Azucena, the Al-tolerant genotype, methylation and expression changes were targeted in genes directly linked to Al exclusion, including organic acid and MATE transporters, whereas BGI showed a broader and less specific epigenomic response.

**Discussion:**

These results suggest that Al stress triggers genotype- and sequence-context-specific epigenomic reprogramming in rice, and that tolerance is associated with a targeted methylation response rather than a diffuse one. This positions DNA methylation as an additional regulatory layer shaping Al tolerance, and pinpoints a short list of candidate genes as priority targets for epigenome-informed breeding strategies in Al-tolerant rice.

## 1 Introduction

Commercial rice, *Oryza sativa* L., is the second most cultivated cereal in the world, covering approximately 167 million hectares globally (Amaya et al., 2019). It is a staple food for over half of the world’s population, providing more than 20% of the calories consumed in regions such as Asia, the Middle East, India, and Latin America (Fukagawa and Ziska, 2019). However, rice productivity is increasingly challenged by biotic and abiotic stresses, which account for approximately 30% and 50% of yield losses worldwide, respectively (Fahad et al., 2017). Among the most important abiotic constraints affecting rice production are drought, salinity, extreme temperatures, and heavy metal toxicity (Wang et al., 2025). Although substantial advances have been made in improving the resistance of current varieties to stressful conditions, a wide range of stresses continues to limit global agricultural production (Zhu, 2016).

Aluminum (Al) toxicity in acidic soils is a major factor limiting rice production, a problem that is worsening due to current fertilization practices, pasture management, and climate change (Fryzova et al., 2018; Kochian et al., 2015; Samac and Tesfaye, 2003; Zheng, 2010). Acid soils, characterized by a pH lower than 5.5, represent nearly 30% of the world’s arable land, with approximately 13% of staple crops cultivated in these areas (Bojórquez-Quintal et al., 2017; Rahman et al., 2018). The most phytotoxic form of Al in acidic soils is the trivalent cation Al³^+^, which rapidly damages root apices within minutes of exposure, particularly affecting the distal transition zone (Bojórquez-Quintal et al., 2017). At the cellular level, Al³^+^ ions inhibit cytoskeletal development, bind to DNA molecules, affecting their compaction and replication, and trigger oxidative stress, impaired nutrient assimilation, and reduced water uptake, collectively resulting in severe inhibition of root elongation (Kochian et al., 2004).

Al tolerance levels vary widely among plant species and genotypes. Rice is considered one of the most Al tolerant cereal crops, significantly more so than sorghum (*Sorghum bicolor*), maize (*Zea mays*), and wheat (*Triticum aestivum*) (Famoso et al., 2010). This characteristic, combined with the wide intraspecific variation in Al tolerance within rice, makes it an excellent model system for investigating the molecular and regulatory mechanisms underlying the contrasting responses to Al stress. Additionally, in rice, genotype-dependent stress responses reflect the combined effects of allelic variation and regulatory divergence, particularly epigenetic changes, prompting our comparison of tolerant versus susceptible genotypes (Pan et al., 2023).

Significant progress has been made in the past decade in elucidating the genomic and transcriptomic mechanisms underlying Al tolerance in plants. In rice, a complex and multifaceted response to Al stress has been described, involving a diversity of genes and regulatory strategies (Famoso et al., 2011; Gallo-Franco et al., 2023; Mustafa and Komatsu, 2016; P. Zhang et al., 2019). Key mechanisms include the exudation of organic acids, particularly citrate and malate, mediated by the ALMT and MATE transporter families, which chelate Al³^+^ in the rhizosphere, as well as internal detoxification through vacuolar sequestration (Kochian et al., 2015). The transcription factor STOP1 has emerged as a central regulator of Al tolerance, controlling the expression of multiple downstream genes involved in organic acid release and proton resistance (Iuchi et al., 2007). Despite these advances, the regulatory layers controlling the transcriptional response to Al stress, particularly at the epigenetic level, remain poorly understood.

Epigenetic modifications, including histone modifications and DNA methylation, represent a key regulatory layer that shapes transcriptional responses without altering the underlying DNA sequence. DNA methylation is the most extensively studied epigenetic mark and occurs in three sequence contexts in plants: CG, CHG, and CHH (where H = A, T, or C), each maintained by distinct enzymatic pathways and associated with different regulatory functions (Law and Jacobsen, 2010). This modification has been implicated in transposable element (TE) silencing, chromosomal interactions, trait inheritance and transcriptional regulation.

Although DNA methylation is considered a relatively stable and transgenerational mark, it is also dynamically responsive to environmental stimuli (Zhang et al., 2018). Stress-induced DNA methylation changes have been documented across a wide range of abiotic stresses in multiple plant species, including drought, salinity, heat, and heavy metal exposure, demonstrating stress-type specificity in methylation responses (Sun et al., 2022). While most stress-induced methylation changes are transient and return to basal levels upon stress removal, some studies have reported short- or long-term epigenetic memory effects, suggesting that DNA methylation may contribute to stress adaptation and transgenerational acclimation (Akhter et al., 2021; Verhoeven et al., 2010). In the context of Al stress, DNA methylation changes have been reported in a small number of plant species, including wheat and common bean, where Al exposure altered global methylation levels and affected the expression of stress-responsive genes (Chang et al., 2020; Sudan et al., 2018). However, no comprehensive genome-wide study linking DNA methylation dynamics to transcriptional regulation under Al stress has been conducted in rice, leaving a critical gap in our understanding of the epigenetic basis of Al tolerance in this globally important crop.

To address this gap, we performed an integrative analysis combining whole-genome bisulfite sequencing (WGBS) and RNA sequencing (RNA-seq) in two *O. sativa* genotypes with contrasting Al responses, the tolerant Azucena (AZU) and the susceptible BGI9311 (BGI), exposed to long-term Al stress conditions. This study aimed to characterize genome-wide DNA methylation changes induced by Al stress, identify differentially methylated regions and their associated genes, and investigate the relationship between methylation dynamics and transcriptional responses in a genotype-comparative framework. Our findings provide the first comprehensive epigenomic map of Al stress responses in rice and offer new insights into the role of DNA methylation in shaping genotype-specific transcriptional programs under Al toxicity.

## 2 Materials and methods

### 2.1 Plant material, growth conditions and aluminum treatment

*The O. sativa* genotypes used in this study were the Al-tolerant Azucena and Al-susceptible BGI9311 (BGI), selected based on their contrasting relative root growth indices under Al stress, as previously characterized by Famoso et al. (2010) and Arbelaez et al. (2017). Seeds were obtained from the Plant Physiology Laboratory at ICESI University, Cali, Colombia, where this contrasting phenotype had also been previously confirmed under the same treatment conditions used in the present study, based on root growth, aluminum-toxicity symptom, and histochemical staining data (Chaura, 2014, unpublished data, **Supplementary Figure 1**).

After surface sterilization and dormancy breaking, the seeds were placed in the dark at 30 °C for 4 days. Germination was then carried out in a culture room at 30 °C under a 12 h light/12 h dark photoperiod for 10 days on agarose medium. Subsequently, the seedlings were transferred to a hydroponic system containing Kimura B nutrient solution (pH 6.0) supplemented with Arnon micronutrients and maintained for 3 weeks. Following this establishment period, the pH of the hydroponic medium was progressively lowered to 4.0 for all plants to establish low-pH basal conditions. For Al stress treatment, plants were exposed to 100 µM AlCl_3_ in the hydroponic medium for 10 days at pH 4.0, while control plants remained in the original hydroponic culture at pH 4.0. Al solubility and speciation were maintained throughout the experiment. At the end of the treatment period, roots were harvested from all plants, immediately frozen in liquid nitrogen, and stored at -80 °C until further use.

### 2.2 DNA and RNA extraction and sequencing

Roots from five individual plants of the same genotype grown under identical conditions were pooled to constitute a single biological replicate. Three independent biological replicates were collected per genotype and treatment condition (control and Al stress). The same biological replicates were used for both DNA and RNA extraction.

Total genomic DNA was extracted using a modified CTAB 2X protocol (Maropola et al., 2015). DNA integrity was assessed by agarose gel electrophoresis and quantified using a NanoDrop spectrophotometer (Thermo Fisher Scientific). Bisulfite sequencing libraries and paired-end (2 × 150 bp) whole-genome bisulfite sequencing (WGBS) were prepared from the extracted genomic DNA by Novogene (Seoul, South Korea) using the Illumina NovaSeq 6000 platform.

Total RNA was extracted using the RNAzol protocol with modifications (**Data Sheet 1**). RNA integrity was assessed by agarose gel electrophoresis and confirmed using Bioanalyzer 2100 (Agilent Technologies). Paired-end (2 × 150 bp) RNA sequencing was performed by Novogene using an Illumina NovaSeq 6000 platform.

### 2.3 Whole-Genome Bisulfite Sequencing (WGBS) read alignment and data imputation

The quality of raw reads was evaluated using FastQC (0.11.9) (Andrews, 2010). Sequencing adapters and low-quality bases (Phred score < 30) were removed using Trimmomatic v0.32 (Bolger et al., 2014). Cleaned reads were mapped to the *Oryza sativa* Nipponbare reference genome (Os-Nipponbare-Reference-IRGSP-1.0 (https://rapdb.dna.naro.go.jp/) from the Rice Annotation Project Database (RAP-DB) using default parameters. Only uniquely aligned reads were retained, and all samples were deduplicated using the Bismark deduplication module. Cytosine-level methylation calls were obtained for CG, CHG, and CHH contexts using METHimpute (Taudt et al., 2018), a Hidden Markov Model-based approach that infers methylation status at individual cytosines even under low sequencing depth or missing data conditions. This approach features a three-state Hidden Markov Model (HMM). The methylation status of a given site was classified as unmethylated (U), methylated (M), or intermediate (I). To avoid poor-quality methylation state calls, only cytosines with a maximum posterior probability of at least 0.99 were retained. All WGBS data processing steps were performed using the MethylStar pipeline (Shahryary et al., 2020).

### 2.4 Genome-wide methylation analysis

Genome-wide methylation patterns were characterized by quantifying the abundance of methylated cytosine (mC) across 100-kb genomic windows for each cytosine context (CG, CHG, and CHH) in all samples. For each window, the difference in mC counts between the Al-stress and control samples was calculated using the average of three biological replicates. Log fold change (LFC) distributions were compared between genotypes using a paired Wilcoxon signed-rank test with Benjamini-Hochberg p-value adjustment.

### 2.5 Identification of Differentially Methylated Regions (DMRs) and differentially methylated genomic features

Base-level methylation state calls were generated using METHimpute, which classifies each cytosine into a discrete methylation state (Unmethylated, Intermediate, or Methylated) based on a Hidden Markov Model (HMM), together with a posterior probability for each call. Differentially methylated regions (DMRs) were then identified using jDMR (Hazarika et al., 2022; Zhou et al., 2025) (https://github.com/jlab-code/jDMRgrid), a heuristic, state-based DMR caller. The genome was partitioned into bins for each cytosine context (CG, CHG, CHH) using the runjDMRgrid function, which automatically determines the optimal bin size per context based on a minimum-cytosine threshold (min.C = 10). only bins meeting this threshold were retained. This yielded bin sizes of 500, 400, and 100 bp for the CG, CHG, and CHH contexts, respectively, with adjacent bins of concordant state subsequently merged into larger regions. The methylation status of each bin was classified as methylated or unmethylated for each sample independently.

Biological replicates (n = 3 per condition) were incorporated via the sample metadata file and processed independently through methimpute and jDMR. State-call matrices for all replicates and bins were generated using makeDMRmatrix and compared pairwise between control (AC) and Al-stress (AT) conditions. Replicate variability was handled through a stringent concordance filter (filterDMRmatrix, replicate.consensus = 1), retaining only bins showing 100% agreement in methylation state among the three biological replicates within each condition. Bins with identical states between conditions (non-polymorphic) were excluded, and only regions showing complete within-group concordance combined with a discordant state between AC and AT were retained as DMRs, classified as hyper- or hypomethylated accordingly. Differential methylation was therefore defined categorically, as a consistent state change between conditions.

The overlap of DMRs with transposable elements (TEs), genes, and promoter regions (2-kb upstream of the transcription start site) was evaluated. DMRs were intersected with each annotated genomic feature using interval overlap analysis, where a DMR was considered associated with a given feature if at least 60% of the DMR length overlapped that feature. DMRs overlapping more than one feature type were retained in each corresponding category independently. Coordinates for these genomic features were obtained from RAP-DB. Chromosome, start, and end positions relative to the *Oryza sativa* IRGSP-1.0 reference genome were used for the overlap analysis.

To assess whether DMRs were preferentially located within transposable elements, upstream regions, and gene bodies, a nucleotide-level overlap analysis was additionally performed. For each DMR dataset (per genotype, sequence context, and methylation direction - hyper or hypomethylated), coordinates were intersected with the corresponding feature coordinates on a per-chromosome basis using the R function data.table::foverlaps, and the number of overlapping base pairs was calculated for each DMR-feature pair. For each feature type, enrichment was tested using a chi-square test on a 2×2 contingency table comparing the number of base pairs located inside versus outside the feature for DMRs against the corresponding proportion expected from the genomic background. Fold enrichment and Cramér’s V were reported as effect-size measures. False discovery rates (FDR) were calculated to correct for multiple testing, and only genomic features with adjusted p-values < 0.01 were considered preferentially enriched for DMRs.

### 2.6 RNA-seq data filtering and analysis

Raw read quality was assessed using FastQC (0.11.9), and adapter sequences and low-quality bases (Phred score < 30) were removed using Trimmomatic v0.32. Cleaned reads were aligned to the Os-Nipponbare-Reference-IRGSP-1.0 genome from the RAP-DB (https://rapdb.dna.naro.go.jp/) using STAR v2.5 (Dobin et al., 2013). Read counts per gene were generated using FeatureCounts v2.0.3 (Liao et al., 2014), selecting the exon counting option and summarizing the results by gene ID. Differential expression analysis was performed using the DESeq2 R package (Love et al., 2014) with default parameters, employing the Wald test for hypothesis testing and the Benjamini-Hochberg (BH) method for multiple testing correction. Differentially Expressed Genes (DEGs) were identified using an adjusted p-value (FDR) < 0.05 based on the BH correction method and an absolute log_2_ fold change (|log_2_FC|) ≥ 2. This stringent fold-change threshold was selected to prioritize genes with large-magnitude, high-confidence expression changes in response to aluminum stress.

### 2.7 Functional enrichment analysis

For each gene identified in the overlaps between DMR-associated gene bodies and upstream regions across CG, CHG, and CHH methylation contexts, and among upregulated (log_2_FC ≥ 2) or downregulated (log_2_FC ≤ −2) genes, functional enrichment analysis was performed using the gprofiler2 R package (Kolberg et al., 2020), querying the *Oryza sativa* annotation database with GO Biological Process (GO: BP), GO Molecular Function (GO: MF), and KEGG pathway databases to identify overrepresented biological processes, molecular functions, and metabolic pathways in the DMR-associated gene body, upstream region, and DEG lists. Significance was assessed at p ≤ 0.05 using the g:SCS (Set Counts and Sizes) multiple-testing correction method implemented in g:Profiler. Unlike standard correction methods such as Bonferroni or Benjamini-Hochberg, which assume independence among tested terms, g:SCS accounts for the hierarchical and correlated structure of Gene Ontology terms. It defines an experiment-wide, species-specific significance threshold based on the distribution of term sizes and their hierarchical relationships, rather than applying a uniform correction across all tests (Reimand et al., 2007). Functional annotations were retrieved from the RAP-DB gene annotation database, and metabolic reactions were obtained from OryzaCyc 9.0.0 (https://pmn.plantcyc.org/organism-summary?object=Oryza) to provide a functional perspective on genes associated with aluminum stress regulation.

### 2.8 Data availability

The raw WGBS data analyzed in this paper can be found in the NCBI Sequence Read Archive (SRA) database under BioProject accession number PRJNA1478175. The raw RNA sequencing data can be found in the NCBI SRA database under BioProject accession number PRJNA980182. Datasets and scripts used for these analyses are available in the GitHub repository: https://github.com/ccsosa/Rice_aluminum_stress_methylated_transcriptome.

## 3 Results

### 3.1 Genome-wide methylation changes associated with Al response in contrasting rice genotypes

Whole-genome bisulfite sequencing (WGBS) was performed on the roots of two contrasting rice genotypes, Al-tolerant Azucena and Al-susceptible BGI, grown under control and Al stress conditions. Sequencing yielded an average of 55 million raw reads per sample, with genome coverage ranging from 88% to 96% and sequencing depths ranging from 14× to 20×. The bisulfite conversion efficiency exceeded 99.5% across all libraries, confirming the reliability of methylation calls for genome-wide analyses. Across both genotypes and treatment conditions, methylated cytosines (mCs) were most abundant in the CG context, followed by CHG and CHH contexts, which is consistent with previously reported methylation patterns in rice genomes (Feng et al., 2016; Gallo‐Franco et al., 2022; Garg et al., 2015; Tan et al., 2016).

**Figure 1:**
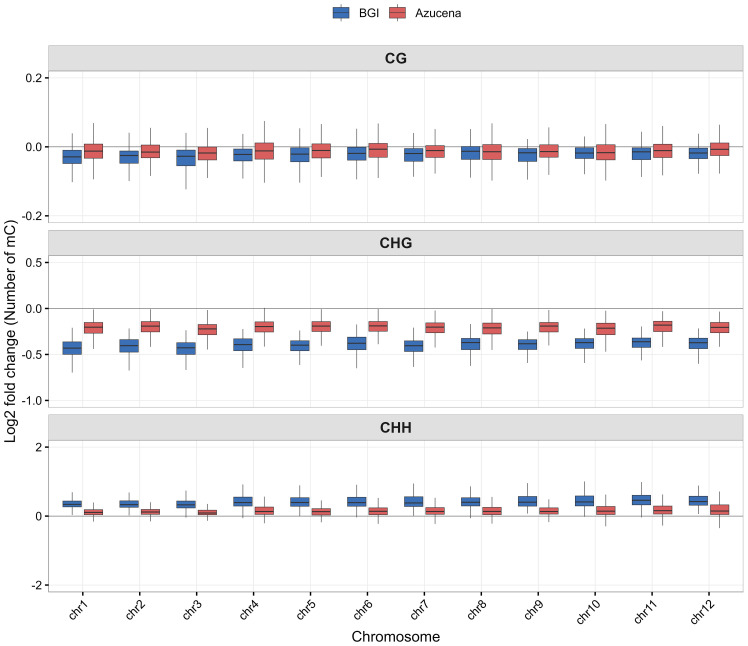
Boxplots showing genome-wide changes in the number of methylated cytosines (mC) abundance under Aluminum (Al) stress in contrasting rice genotypes Azucena (Al-tolerant) and BGI (Al-sensitive), per chromosome. The y-axis shows the Log Fold Change (LFC) in mC counts (Al stress relative to control) across 100-kb genomic windows, calculated separately for the CG, CHG, and CHH methylation contexts (where H = A, T, or C), in BGI (blue) and Azucena (red).

To characterize the genome-wide methylation responses to Al stress, we quantified changes in mC abundance across 100-kb genomic windows for each cytosine context (CG, CHG, and CHH) and compared the log fold change (LFC) distributions between BGI and Azucena across all chromosomes (**Figure 1**). Chromosome-specific distributions are shown in **Supplementary Figure 2**. To further characterize this pattern, we analyzed the distribution of mean methylation differences (Control - Treatment) across 100-kb windows overlaid with the genomic positions of high-confidence DMRs for all chromosomes (**Supplementary Figures 3**–**5**).

CG methylation was largely stable under Al stress in both genotypes, with median LFC values close to zero in both BGI (-0.026) and Azucena (-0.013), indicating broad maintenance of CG methylation patterns under stress. Despite this overall stability, LFC distributions differed significantly between genotypes across all contexts (paired Wilcoxon test, adjusted *p* < 2.2e-16), indicating subtle but statistically detectable genotypic differences, even in the most stable methylation context.

CHG methylation showed a pronounced and consistent genome-wide reduction under Al stress, with a markedly stronger effect in the susceptible genotype. The median LFC values reached -0.41 in BGI versus -0.21 in Azucena (median difference between genotypes = 0.12, adjusted *p* < 2.2e-16), and this hypomethylation pattern was uniform across all chromosomes, suggesting widespread disruption of CHG methylation maintenance in response to Al exposure.

The most remarkable genotype-dependent contrast was observed in the CHH context. BGI exhibited a clear genome-wide gain in CHH-methylated cytosines under Al stress (median LFC = 0.41), whereas Azucena showed less hypermethylation (median LFC = 0.16, adjusted *p* < 2.2e-16). Taken together, Al stress induced opposing context-specific methylation dynamics and global CHG hypomethylation concurrent with CHH hypermethylation, with substantially stronger responses in the Al-susceptible BGI genotype, suggesting that epigenomic perturbation under Al exposure is more extensive in the susceptible background.

### 3.2 Al stress-induced DMRs in contrasting genotypes

**Figure 2:**
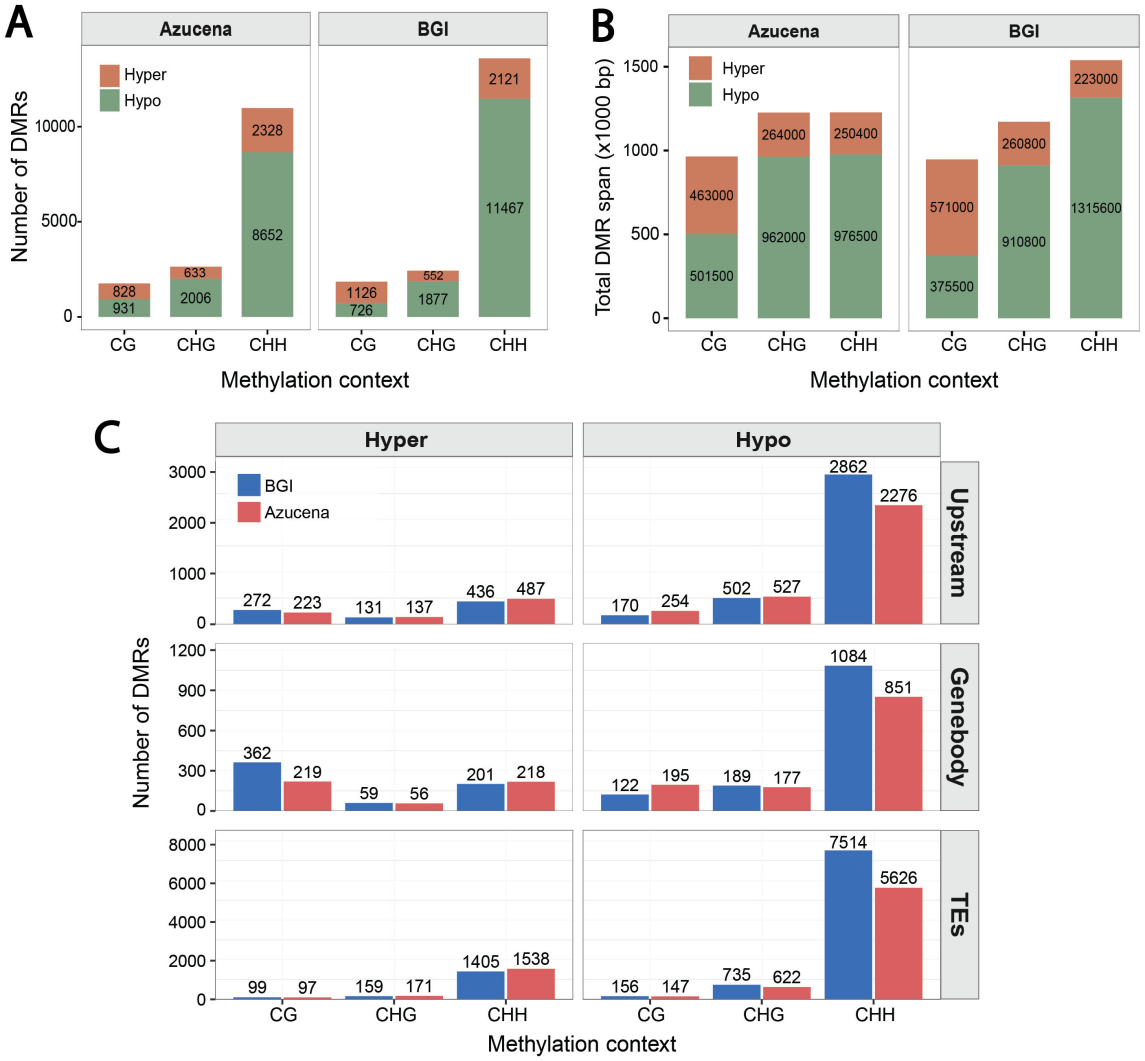
Genome-wide distribution and genomic annotation of Differentially Methylated Regions (DMRs) in response to Aluminum stress in the rice genotypes Azucena (Al-tolerant) and BGI (Al-sensitive). DMRs were identified separately for the three cytosine sequence contexts: CG, CHG, and CHH (where H = A, T, or C). **(A)** Number of hyper- and hypomethylated DMRs identified in Azucena and BGI following aluminum exposure in each methylation context. **(B)** Total genomic span (bp) covered by DMRs in each methylation context. **(C)** Genomic distribution of DMRs across three annotation categories: upstream regulatory regions (2 kb upstream of the transcription start site), gene bodies, and transposable elements (TEs), shown separately for each methylation context.

To identify specific genomic regions undergoing methylation changes in response to Al stress, we performed DMR analysis by comparing control and stress conditions in both genotypes (**Figure 2A**). DMRs were initially detected using context-specific genomic windows (500 bp for CG, 400 bp for CHG, and 100 bp for CHH methylation) and subsequently merged when adjacent differentially methylated windows occurred in continuous genomic regions, generating DMRs of varying lengths. Across all cytosine contexts, hypomethylated DMRs predominated over hypermethylated DMRs in both BGI and Azucena, suggesting that the local loss of DNA methylation represents the most common epigenomic response to Al exposure (**Figure 2A**).

The CHH context exhibited the highest number of DMRs. In Azucena, 10980 CHH-DMRs were identified (2,328 hypermethylated and 8,652 hypomethylated), compared with 2639 CHG-DMRs (633 hypermethylated and 2,006 hypomethylated) and 1759 CG-DMRs (828 hypermethylated and 931 hypomethylated). BGI displayed an even stronger response, with 13588 CHH-DMRs (2121 hypermethylated and 11467 hypomethylated), 2429 CHG-DMRs (552 hypermethylated and 1877 hypomethylated), and 1852 CG-DMRs (1126 hypermethylated and 726 hypomethylated). Across all sequence contexts, BGI consistently showed a greater number of DMRs than Azucena, particularly in the CHH context, supporting a more extensive epigenomic response in the Al-sensitive genotype.

Because adjacent DMR windows were merged into continuous regions, we further evaluated the cumulative genomic span covered by DMRs and the DMR-length distributions to determine whether the larger methylation-remodeled genomic space observed in BGI was due to larger DMRs or a greater number of affected loci. The mean DMR lengths closely reflected the minimum window sizes used for DMR detection due to the merging of neighboring DMRs (BGI-CG: 511pb, BGI-CHG:444 pb, BGI-CHH: 113 pb; Azucena-CG: 548 pb, Azucena-CHG: 505 pb, Azucena-CHH: 112 pb). Although some merged regions extended up to 5.2 kb, most DMRs remained close to the minimum detection size. Importantly, average DMR lengths were highly similar between genotypes within each sequence context. The complete DMR length statistics for all contexts and methylation states are provided in **Supplementary Table 1**. Analysis of the cumulative genomic span covered by DMRs revealed a trend similar to that of the number of DMRs (**Figure 2B**). CHH-DMRs accounted for the largest fraction of methylation-remodeled genomic space in both genotypes, even with the lowest size of DMR (100bp), spanning approximately 1.23 Mb in Azucena and 1.54 Mb in BGI. In all three sequence contexts, hypomethylated DMRs represented most of the affected genomic span, indicating that methylation loss occurred more frequently and across a larger proportion of the genome than methylation gains (**Figure 2B**). Interestingly, BGI in the CG context was the only one with a higher number of DMRs and genome coverage.

Interestingly, the DMR landscape presented an apparent contrast to the genome-wide trends reported in Section 3.1 (**Figure 1**). While CHH methylated cytosine abundance increased broadly across chromosomes under Al stress, mostly in BGI, the majority of discrete CHH-DMRs corresponded to hypomethylated regions. This contrast is evident when the mean methylation difference (Control - Treatment) per 100-kb window is plotted against genomic position and overlaid with the coordinates of DMRs across all twelve chromosomes (**Supplementary Figure 5**). The genome-wide signal remains consistently and substantially negative throughout the rice genome, reflecting the large-magnitude, genome-wide gain in CHH methylation under stress, whereas the CHH-DMRs showed a marked trend towards hypomethylation in both genotypes.

### 3.3 Genomic distribution of Al stress-induced DMRs

To characterize the genomic distribution of DMRs, their overlap with upstream regulatory regions (2 kb), gene bodies, and transposable elements (TEs) was assessed, requiring a minimum overlap of 60% of the DMR length with the annotated feature (**Figure 2C**). DMRs were distributed across all three genomic features, with clear differences in abundance according to cytosine context, methylation direction, and genotype. CHH hypomethylated DMRs were the most abundant class across all features in both genotypes, with BGI consistently showing higher counts than AZU. The largest accumulation was observed in TEs, where BGI harbored 7514 and AZU 5626 CHH hypomethylated DMRs. In upstream regulatory regions, these numbers reached 2862 and 2276, respectively, and within gene bodies, 1084 and 851. DMRs in the CG and CHG contexts were considerably less abundant and showed smaller genotypic differences across all the features.

To determine whether these raw counts reflected positional enrichment rather than the relative genomic size of each feature class, we performed a nucleotide-level enrichment analysis relative to genomic background for each feature, context, methylation direction, and genotype (**Supplementary Table 2**). This analysis confirmed that DMR enrichment within TEs was context-dependent: CHH- and CHG-context DMRs were significantly enriched in TEs in both genotypes (fold enrichment = 1.05-1.65; chi-square test, all p-adj < 2.66e-15), whereas CG-context DMRs were only enriched in TEs for hypo-DMRs in BGI (fold enrichment = 0.64-1.08). DMRs were also significantly enriched in upstream regulatory regions across nearly all contexts and genotypes (fold enrichment = 1.04-1.40, p-adj < 2.66e-15), with the exception of BGI CHH-hyper DMRs. In contrast, DMRs were consistently and strongly depleted within gene bodies across all contexts, methylation directions, and genotypes (fold enrichment = 0.29-0.89).

The proportional distribution of DMRs across genomic features revealed that TE-associated DMRs represented the largest fraction of methylation changes in both genotypes (**Figure 2C**). TEs accounted for 56-61% of CG-DMRs, 97-100% of CHG-DMRs, and 51-55% of CHH-DMRs, with CHH hypomethylated DMRs representing 55.3% and 51.2% of all CHH-DMRs in BGI and AZU, respectively. Genome-wide studies have revealed frequent promoter-proximal methylation changes in stress-responsive genes (Abubakar et al., 2023), which is consistent with our finding that upstream genomic regions comprise 21-27% of DMRs, whereas gene bodies represent the smallest fraction at 8-26%. The particularly strong enrichment of CHG-DMRs within TEs, approaching 100% in both genotypes, suggests that repetitive elements are the primary targets of Al-induced CHG methylation remodeling. The complete proportional distribution of DMRs across all sequence contexts, methylation states, and genotypes is presented in **Supplementary Table 3**.

### 3.4 Functional characterization of DMR-associated genes

To explore the biological significance of Al stress-induced methylation changes, functional enrichment analyses were performed on genes associated with DMRs in gene bodies and upstream (2kb) regulatory regions in both genotypes. In total, 1717 and 2017 gene body-associated DMR genes were identified in AZU and BGI, respectively, while 3905 and 4374 upstream-associated DMR genes were identified for AZU and BGI, respectively, suggesting a broader genomic footprint of upstream methylation changes relative to gene body changes.

To identify conserved epigenetic responses shared between genotypes, we compared gene body- and upstream-associated DMRs that required concordance in both sequence context and methylation direction. This analysis identified 199 common gene body-associated DMR genes and 363 common upstream-associated DMR genes. Functional enrichment of these shared gene sets revealed enrichment for the KEGG pathway *sesquiterpenoid and triterpenoid biosynthesis* (eight genes), suggesting that secondary metabolite pathways related to terpenoid production represent a conserved epigenetic target of Al stress across both tolerant and susceptible genotypes.

**Figure 3:**
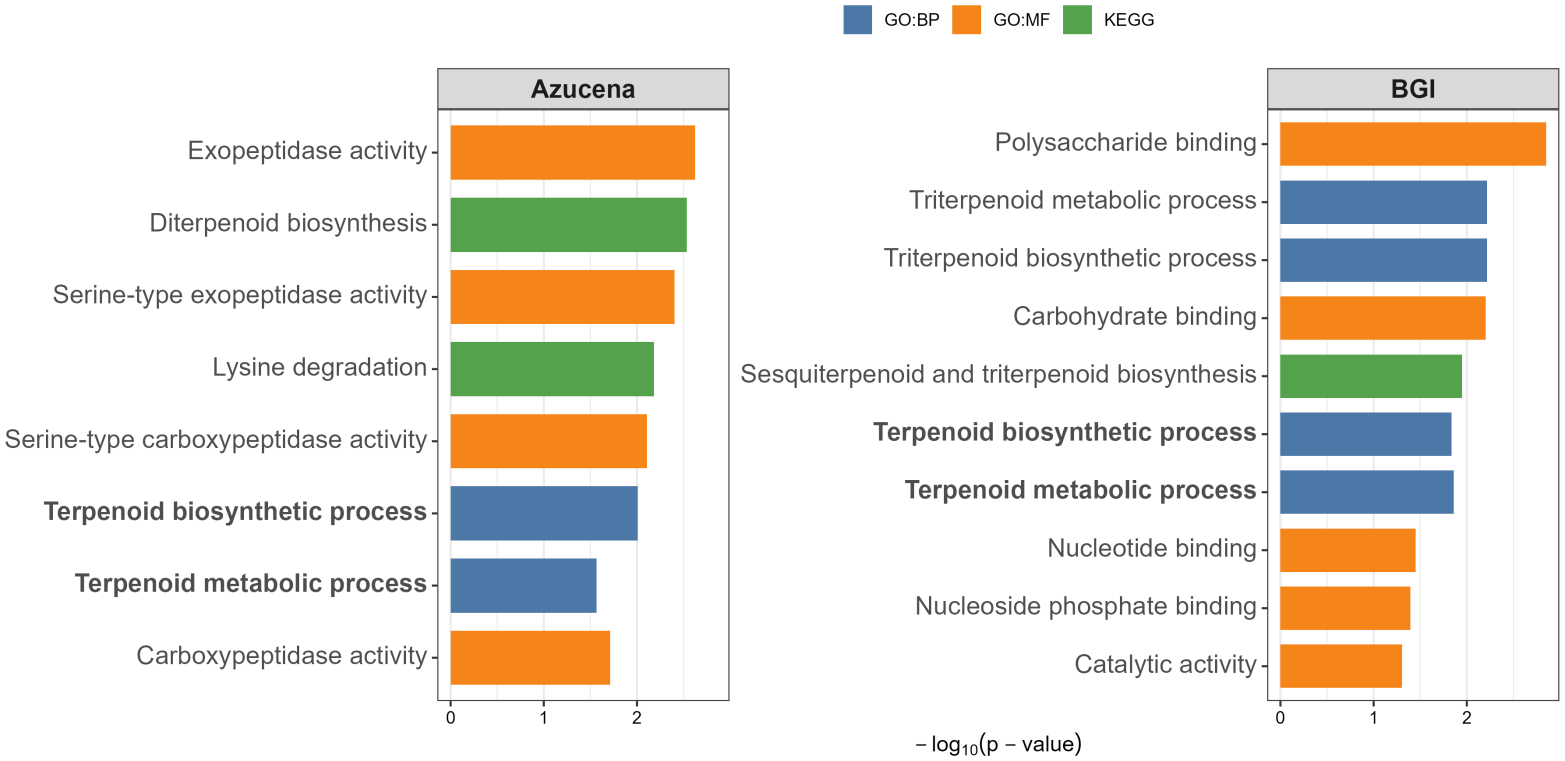
Functional enrichment of genes associated with gene-body Differentially Methylated Regions (DMRs) in Azucena and BGI genotypes. Bold terms indicate categories shared between both rice genotypes. Values are expressed as -log_10_ (adjusted p-value), obtained using gprofiler2 for enriched Gene Ontology Biological Process (GO: BP), and Molecular function (GO: MF) terms, and KEGG metabolic pathways.

Functional enrichment analyses were performed independently on the genotype-exclusive DMR-gene sets, genes associated with DMRs found only in AZU or only in BGI, for both gene bodies and upstream regions (**Supplementary Table 4**). No significant enrichment was detected for upstream-associated DMR genes in either of the genotypes. For gene body-associated DMRs, both shared functional categories and genotype-specific enrichments were identified (**Figure 3**), revealing that while the two genotypes differ in the specific genes undergoing methylation changes, they partially converge on common biological pathways.

Notably, the GO terms *terpenoid biosynthetic process* (GO:0016114) and *terpenoid metabolic process* (GO:0006721) were significantly enriched in the genotype-specific gene sets of both AZU and BGI (bold terms in **Figure 3**), indicating that stress-induced methylation changes in gene bodies converge on terpenoid secondary metabolism pathways in both genotypes, despite involving distinct gene subsets. This convergence suggests that the epigenetic regulation of terpenoid metabolism represents a shared but genotype-independent response to Al stress.

In addition to this shared signal, important genotype-specific differences were observed. In AZU, the enrichment profile was predominantly associated with proteolytic and peptidase-related molecular functions, including *exopeptidase*, *serine-type exopeptidase*, *serine-type carboxypeptidase*, and *carboxypeptidase activities*. KEGG analysis additionally identified enrichment in *diterpenoid biosynthesis* and *lysine degradation* pathways. Together, these results suggest that methylation changes in AZU gene bodies preferentially affect pathways associated with protein turnover, peptide processing, and diterpenoid-related secondary metabolism, which may contribute to a more targeted and regulated stress response in the tolerant genotype.

In contrast, BGI showed enrichment for more specific terpenoid pathways, *triterpenoid metabolic processes*, *triterpenoid biosynthetic processes*, and the KEGG pathway *of sesquiterpenoid and triterpenoid biosynthesis*, alongside molecular functions related to carbohydrate and polysaccharide binding, such as *polysaccharide binding*, *carbohydrate binding*, *nucleotide binding*, *nucleoside phosphate binding*, and *catalytic activity*. The broader and more diverse enrichment profile observed in BGI, spanning terpenoid metabolism, carbohydrate-interacting functions, and general enzymatic activity, is consistent with a less selective and more extensively perturbed epigenomic response in the susceptible genotype.

### 3.5 Methylation and transcriptional changes under Al stress

**Figure 4:**
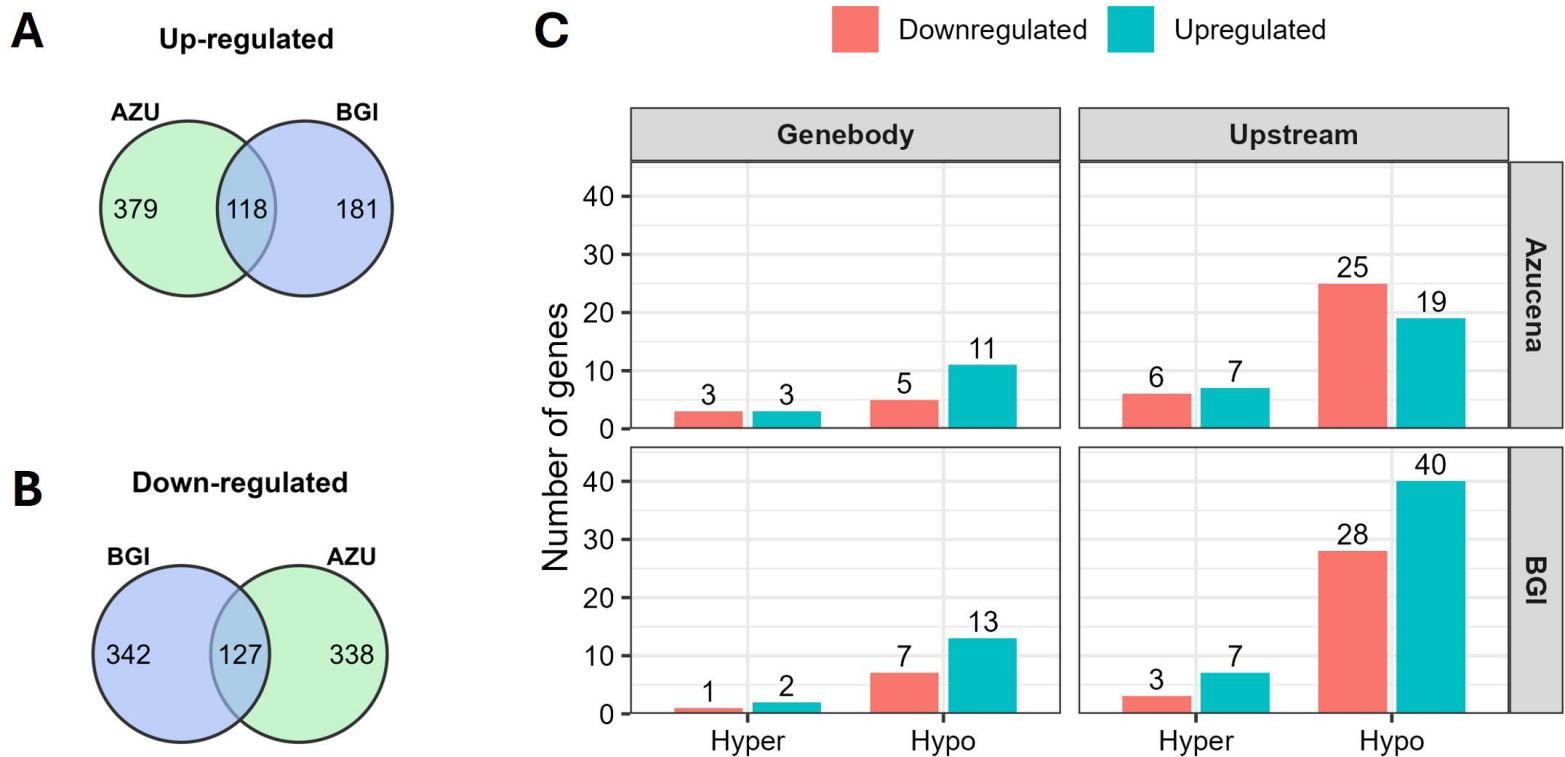
**(A)** Venn diagram showing unique and shared up-regulated Differentially Expressed Genes (DEGs) between the Al-tolerant Azucena and Al-susceptible BGI genotypes under Al stress. **(B)** Venn diagram showing unique and shared down-regulated DEGs between Azucena and BGI under Al stress. **(C)** Bar plots showing the number of DEGs exhibiting coordinated changes in DNA methylation and gene expression (i.e., hypomethylation paired with up-regulation, or hypermethylation paired with down-regulation), distributed by genomic feature (gene body or upstream region, 2 kb upstream of the transcription start site), cytosine context (CG, CHG, and CHH, where H = A, T, or C), and methylation direction (hyper- or hypomethylated), in AZU and BGI.

To characterize the transcriptional response to Al stress, differential expression analysis was performed for both genotypes using a threshold of |log_2_FC| ≥ 2 and an adjusted p-value < 0.05. Log_2_FC values ranged from -7.9 to 8.7 in Azucena and from -7.3 to 7.4 in BGI. A total of 962 DEGs were identified in Azucena (497 upregulated and 465 downregulated), compared to 768 DEGs in BGI (299 upregulated and 469 downregulated). Azucena showed a higher proportion of upregulated genes, whereas BGI was characterized by a predominance of downregulated genes, suggesting divergent transcriptional strategies between the tolerant and susceptible genotypes under Al stress. A total of 118 upregulated and 127 downregulated genes were shared between Azucena and BGI (**Figures 4A, B**), indicating the presence of both conserved and genotype-specific transcriptional responses.

To evaluate the relationship between DNA methylation and gene expression, DMR-associated genes were intersected with DEG datasets. In total, 71 genes in Azucena and 93 genes in BGI exhibited both differential methylation and expression under Al stress conditions (**Supplementary Table 5**). Most genes showing these coordinated changes were associated with CHH-context DMRs located in the upstream regulatory regions (**Figure 4C**), rather than with gene body methylation changes. Functional enrichment analysis of these methylated-differentially expressed genes did not yield any significantly enriched GO terms or KEGG pathways in either genotype.

Among the 71 genes in Azucena and 93 genes in BGI showing both methylation and expression changes, only three genes were shared between both genotypes: Os01g0566500 (CCD8A, dioxygenase involved in carlactone/strigolactone biosynthesis), Os04g0556400 (UGT93B9, UDP-glycosyltransferase involved in cytokinin glucosylation), and Os06g0486900 (FDH2, mitochondrial formate dehydrogenase), indicating that the epigenetic-transcriptional response to Al stress is largely genotype-specific (**Table 1**). Remarkably, all three shared genes showed opposite transcriptional responses between genotypes despite overlapping methylation changes, with Azucena exhibiting repression and BGI activation in all cases, further underscoring the genotype-dependent nature of the methylation-expression relationship under Al stress. The complete list of genes with coordinated methylation and expression changes is provided in **Supplementary Table 5**.

**Table 1:** Shared genes exhibiting both differential methylation and differential expression in both Azucena and BGI rice genotypes after 10 days exposure to Al stress.

Gene ID/name	Annotation	Genotype	Context	Gene location	Methylation status	Expression status
Os01g0566500 (CCD8A)	Dioxygenase RAMOSUS1, carlactone/strigolactone biosynthesis	Azucena	CG/CHH	Upstream	Hypo	Down
		BGI	CHH	Upstream	Hypo	Up
Os04g0556400 (UGT93B9)	UDP-glycosyltransferase, cytokinin glucosylation	Azucena	CHH	Gene body	Hyper	Down
		BGI	CHH	Gene body	Hypo	Up
Os06g0486900 (FDH2)	Formate dehydrogenase, mitochondrial	Azucena	CHG	Gene body	Hypo	Down
		BGI	CHG/CG	Upstream/Gene body	Hyper/Hypo	Up

Name and annotation were obtained from the Rice Annotation Project Database (RAPDB). The methylation status indicates whether the gene or regulatory region is hypo-or hypermethylated. The context shows the methylation context of CG, CHG, and CHH (where H = A, T, or C). Gene location shows whether it is a gene body or upstream region (2 kb upstream of the transcription start site) and expression status shows whether a gene is upregulated or downregulated.

Functional analysis of the genotype-exclusive differentially methylated and expressed genes showed several candidates with potential relevance to Al stress responses (**Table 2**). Among these, genes encoding transporters involved in organic acid metabolism and transport were detected, including a C4-dicarboxylate/malate transporter (Os05g0219900) and members of the MATE transporter family (Os10g0345100, Os08g0480000), which are notable because organic acid efflux is a well-established mechanism of Al detoxification in plants. Several genes associated with oxidative stress responses were also identified, including glutathione S-transferases (Os10g0481300, Os01g0369700), peroxidases (Os04g0688500), aldehyde oxidases (Os07g0281700), and oxalate oxidases (Os03g0693900), suggesting that methylation changes may affect pathways involved in reactive oxygen species detoxification and redox homeostasis under Al stress.

**Table 2:** Functional annotation of selected differentially methylated and expressed genes associated with Azucena and BGI rice contrasting genotypes under 10 days exposure to Al stress.

Gene ID	Genotype	Gene annotation	Description	Meth status	Sequence context	Gene location	Expression status
Os01g0369700 (OsGSTF5)	Azucena	Phi-class Glutathione S-transferase (GST)	Glutathione S-transferase is involved in detoxification and protection against oxidative stress.	Hypo	CG	Gene body	Up
Os04g0688500 (OsPRX62)		class III peroxidase enzyme	Class III peroxidases are associated with ROS scavenging, defense responses, and stress adaptation.	Hypo	CHH	Upstream	Up
Os03g0693900 (OsOxO3)		Germin-like protein (GLP)	Oxalate oxidase is involved in plant defense and reactive oxygen species production.	Hypo	CHH	Upstream	Up
Os03g0571900 (OsMATE14)		Multidrug and Toxic Compound Extrusion (MATE) transporter	MATE transporters are involved in phenolic compound transport and mineral homeostasis.	Hypo	CHH	Gene body	Up
Os04g0401700 (OsHAK1)		High-Affinity Potassium Transporter 1	High-affinity potassium transporter required for K^+^ uptake under nutrient limitation and abiotic stress.	Hypo	CHH	Upstream	Up
Os05g0101600 (CYP722B1)		Cytochrome P450 family	Cytochrome P450 is involved in secondary metabolism, hormone biosynthesis, and stress responses.	Hypo	CHH	Upstream	Up
Os02g0503700 (CYP81L5)		Cytochrome P450 family	Cytochrome P450 is involved in secondary metabolism, hormone biosynthesis, and stress responses.	Hypo	CG	Gene body	Up
Os05g0219900 (SLAH4)	BGI	Slow Anion Channel-Associated (SLAH) protein	Anion transporters are involved in ion homeostasis and adaptation to abiotic stress.	Hyper	CHH	Upstream	Down
Os10g0345100 (OsMATE40)		Multi-Antimicrobial Extrusion (MATE) family protein	MATE family transporters are potentially involved in metabolite transport and stress adaptation.	Hypo	CHH	Upstream	Up
Os08g0480000 (OsMATE28)		Multi-Antimicrobial Extrusion (MATE) family protein	MATE family transporters are potentially involved in metabolite transport and stress adaptation.	Hypo	CHH	Gene body	Up
Os10g0481300 (OsGSTU47)		tau-class glutathione S-transferase (GST)	Glutathione S-transferase is involved in ROS detoxification and antioxidant defense.	Hypo	CHH	Upstream	Down
Os07g0281700 (OsAO3)		abscisic-aldehyde oxidase	Aldehyde oxidase is involved in ABA-related stress signaling and osmotic stress responses.	Hypo	CHH	Gene body	Up
Os05g0537100 (OsWRKY7)		WRKY transcription factor 7	WRKY transcription factors regulate defense and stress-responsive gene expression.	Hyper	CHH	Upstream	Down
Os08g0437300		R2R3-MYB family transcription factor	It acts as a potential negative regulator of stress tolerance, showing differential expression in response to low temperatures during germination.	Hypo	CHH	Gene body	Up
Os07g0558100 (OsMYB41)		MYB transcription factor	The MYB transcription factor is involved in root suberization and cell wall modification.	Hypo	CHH	Gene body	Up
Os10g0562900 (OsERF096)		AP2/ERF-domain family transcription factor	AP2/ERF transcription factors are involved in abiotic stress signaling and hormone responses.	Hypo	CHH	Upstream	Down
Os08g0338500 (OsPMEI30)		Pectinesterase Inhibitor (PMEI) domain-containing protein	Pectin methylesterase inhibitors regulate cell wall structure and pectin modification.	Hypo	CHH	Gene body	Up
Os07g0418500 (CYP72A1)		cytochrome P450 72A1	Cytochrome P450 is involved in defense signaling, detoxification, and hormone metabolism.	Hypo	CHH	Gene body	Up
Os11g0289700 (CYP87B1)		Cytochrome P450 family protein	Cytochrome P450 is involved in defense signaling, detoxification, and hormone metabolism.	Hypo	CHH	Upstream	Down
Os09g0530300		Cytochrome P450 family of proteins.	Cytochrome P450 is associated with secondary metabolism and stress-related biosynthetic pathways.	Hypo	CHH	Upstream	Up
Os10g0167200		Cytochrome P450 family of proteins.	Cytochrome P450 is associated with secondary metabolism and stress-related biosynthetic pathways.	Hypo	CHH	Upstream	Up
Os10g0513900		Conserved hypothetical protein.	Cytochrome P450 is associated with secondary metabolism and stress-related biosynthetic pathways.	Hypo	CHH	Upstream	Down

Gene annotation, Gene name, and Description were obtained from RAPDB. Methylation status shows whether the gene or regulation region was hypo-or hypermethylated, and Sequence Context shows the methylation context CG, CHG, and CHH (where H = A, T, or C). Gene location shows whether it is a gene body or upstream region, and expression status shows whether a gene is upregulated or downregulated.

In addition, multiple transcriptional regulators were associated with DMRs, including members of the WRKY (Os05g0537100), MYB (Os08g0437300, Os07g0558100), and AP2/ERF transcription factor families (Os10g0562900), indicating the potential epigenetic regulation of stress-responsive gene networks. Genes related to cell wall modification were also detected (Os03g0571900-Phenolics efflux transporter and Os04g0401700-Potassium transporter), notably pectin methylesterases, and their inhibitors are emerging as major targets of stress-responsive epigenetic regulation (Zhang et al., 2022), consistent with our finding that a pectin methylesterase inhibitor (Os08g0338500) is a DMR-associated gene. Finally, several genes involved in terpenoid biosynthesis, including diterpene cyclases and cytochrome P450 enzymes (Os05g0101600, Os02g0503700, Os07g0418500, Os11g0289700, Os09g0530300, Os10g0167200, and Os10g0513900), were identified among the DMR-associated loci, consistent with the enrichment of terpenoid metabolic pathways observed in the functional enrichment analysis. Together, these results indicate that Al-induced methylation changes affect genes involved in organic acid transport, oxidative stress mitigation, cell wall remodeling, transcriptional regulation, and secondary metabolism, all of which have been previously implicated in plant adaptation to Al stress.

## 4 Discussion

This study provides the first comprehensive genome-wide characterization of DNA methylation dynamics and their relationship with gene expression under Al stress in rice, integrating WGBS and RNA-seq data from two genotypes with contrasting Al tolerance phenotypes. Our results revealed that Al stress triggers extensive context-specific epigenomic reprogramming that differs between the tolerant genotype Azucena and the susceptible BGI, establishing a direct link between genotype-dependent methylation dynamics and transcriptional divergence under Al toxicity. Together, these findings suggest that epigenetic regulation represents an additional layer of control over Al stress adaptation in rice, operating in parallel with the transcriptional and genomic mechanisms previously described (Famoso et al., 2011; Gallo-Franco et al., 2023).

### 4.1 Different methylation contexts drive specific and contrasting physiological responses

Evidence suggests that methylation contexts are established and maintained by distinct molecular pathways, each with different regulatory implications. In plants, CG methylation is broadly maintained by MET1 and is largely context-independent, whereas CHG methylation depends on CMT3/ZMET2 via recognition of H3K9me2, tightly linking it to heterochromatin and transposon silencing (Fang et al., 2022; Gouil and Baulcombe, 2016). CHH methylation is primarily established and maintained through the RNA-directed DNA methylation (RdDM) pathway involving DRM2, making it the most environmentally responsive of the three contexts (Kenchanmane Raju et al., 2019; Law and Jacobsen, 2010; López et al., 2022). Consistent with these pathway-specific properties, our results suggest that the three cytosine contexts responded differently to Al stress in both magnitude and direction.

CG methylation remained largely stable under Al stress in both genotypes, with median LFC values close to zero, consistent with the constitutive and robust maintenance of CG methylation by MET1. In contrast, CHG methylation showed a pronounced and uniform genome-wide reduction in both genotypes, with a significantly stronger effect in the susceptible BGI (median LFC = -0.41) than in the tolerant Azucena (median LFC = -0.21). This pattern aligns with previous reports showing that CHG methylation is more susceptible to environmental perturbations than CG methylation (Boyko and Kovalchuk, 2008) and is consistent with studies reporting preferential CHG hypomethylation under heavy metal stress in plants (Ou et al., 2012). A plausible mechanism underlying the observed CHG hypomethylation could be the production of reactive oxygen species (ROS), a well-documented consequence of Al³^+^ toxicity in root cells (Panda and Matsumoto, 2007; Shahid et al., 2014). ROS can induce DNA damage, including double-strand breaks, which may interfere with the capacity of DNA to serve as a methyl group acceptor, resulting in passive methylation loss at affected sites (Aina et al., 2004; Jing et al., 2022; Ou et al., 2012). Similarly, the interaction between ROS production and stress-induced DNA methylation changes has been proposed as a driver of stress memory and acclimation, offering a framework for understanding how Al stress may trigger sustained epigenetic reprogramming in rice (Abdulraheem et al., 2026).

**Figure 5:**
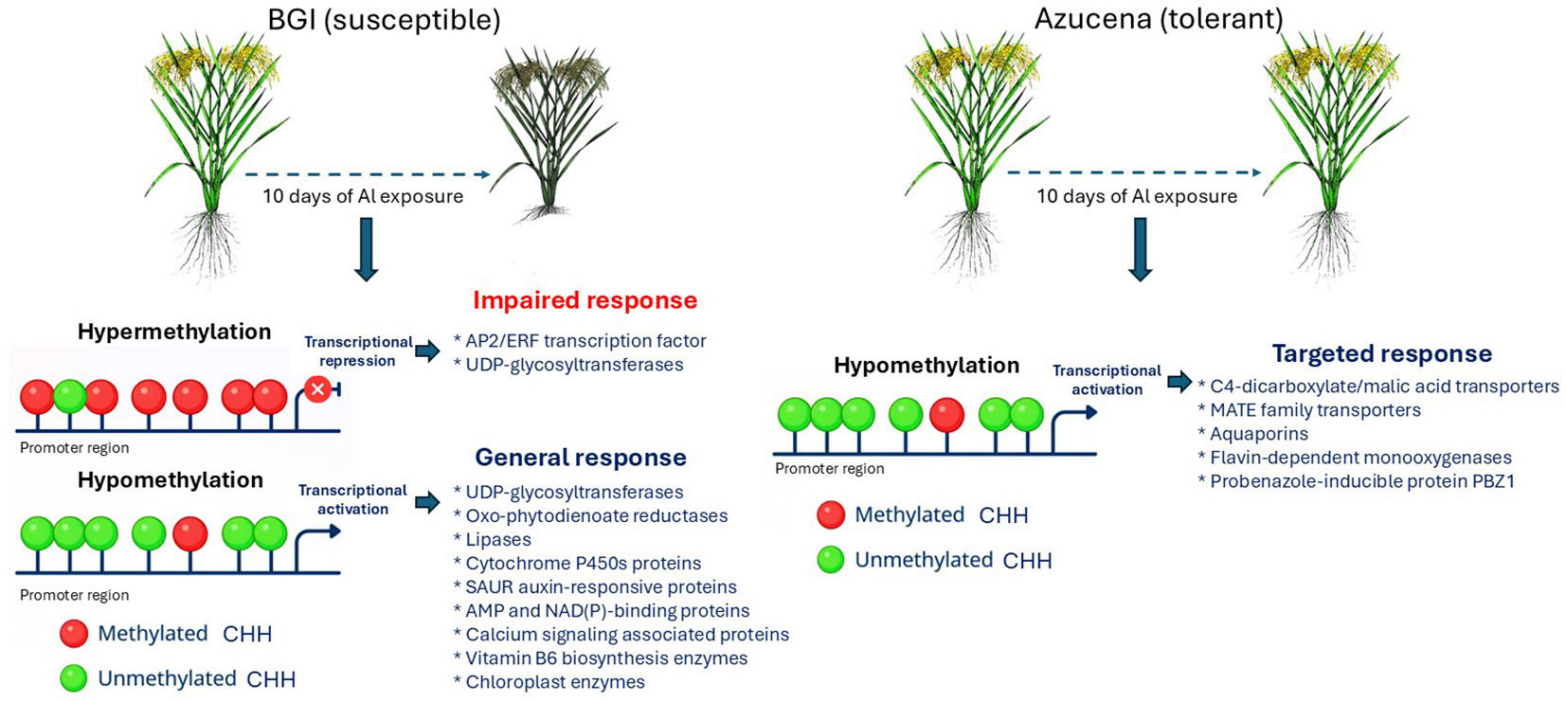
Schematic model summarizing genotype-specific transcriptional and epigenetic responses to 10 days of aluminum (Al) exposure in the Al-susceptible BGI and Al-tolerant Azucena rice genotypes. At the level of coordinated methylation-expression changes, Azucena and BGI shared only three differentially methylated and expressed genes, all exhibiting opposite transcriptional modulation, highlighting that Al stress responses are strongly dependent on the genetic background. Azucena orchestrates an epigenetically enabled Al tolerance program centered on the activation of organic acid transporters, MATE family members, aquaporins, and ROS-regulatory enzymes, whereas BGI displays a broader and less specific response marked by the activation of general stress associated genes and the repression of key transcriptional regulators, limiting its response to stress conditions.

Stress-induced DNA methylation is known to be highly context-specific, with CHH sites and transposable elements frequently targeted, a pattern that is broadly conserved across plant abiotic stress responses (Abdulraheem et al., 2024). The CHH context showed a contrasting and genotype-dependent response: BGI exhibited a clear genome-wide gain in CHH methylation (median LFC = 0.41), whereas Azucena showed only modest changes (median LFC = 0.16). This divergence in CHH dynamics between genotypes is particularly informative because CHH methylation is maintained by the RdDM pathway, which is sensitive to small RNA availability and chromatin accessibility. The stronger CHH hypermethylation in BGI may reflect a more pronounced activation of RdDM as a genome defense mechanism in response to the more extensive epigenomic perturbation observed in the susceptible genotype, potentially as an attempt to silence TEs that become active under stress (Hossain et al., 2017). In contrast, the more restrained CHH response in Azucena may reflect a more precise and targeted epigenomic regulation that avoids widespread disruption while still enabling locus-specific methylation changes in functionally relevant genes (**Figure 5**).

Beyond its role in TE silencing and considering that current evidence is opposing, the global CHH hypermethylation observed in both genotypes, with a strong emphasis on BGI, may also represent a direct DNA-protective function. We hypothesize that methylated cytosines may be less susceptible to oxidative damage than unmethylated cytosines and may shield the genome from ROS-induced DNA damage, a well-documented consequence of Al³^+^ toxicity in root cells (Aina et al., 2004; Jing et al., 2022; Ou et al., 2012). Under this interpretation, CHH hypermethylation may represent a dual protective response: reinforcing TE silencing through RdDM while simultaneously reducing the vulnerability of cytosine residues to oxidative damage.

### 4.2 Spatial heterogeneity of Al stress-induced methylation changes

An interesting result of the Al stress epigenetic response in rice is the spatial heterogeneity of methylation changes across the genomic scale. At the local level, the DMR landscape revealed an apparent contrast to these genome-wide trends. While CHH methylation increased broadly across chromosomal domains under Al stress, most prominently in BGI, the majority of discrete CHH-DMRs corresponded to hypomethylated regions. This divergence can be reconciled by considering the spatial scale of each analysis: the genome-wide window approach captures diffuse, low-magnitude gains distributed across large chromosomal domains, whereas the DMR analysis identifies discrete loci with statistically robust directional changes. Thus, Al stress appears to simultaneously drive large-scale, diffuse gains in CHH methylation across the genome, alongside targeted losses at specific loci, a spatially heterogeneous remodeling of the asymmetric methylation landscape rather than a uniform directional shift. The global CHH hypermethylation observed in BGI may therefore reflect not only a passive consequence of epigenomic perturbation, but also an active genome defense response mediated by the RdDM pathway, aimed at reinforcing TE silencing under stress conditions, a mechanism previously proposed in the context of heavy metal and Al stress responses in plants (Gallo-Franco et al., 2020). Such dual patterns combining diffuse hypermethylation with locus-specific hypomethylation have been reported under other abiotic stress contexts and may reflect the opposing and spatially segregated activities of the RdDM and active demethylation pathways operating simultaneously under stress (López et al., 2022; Sun et al., 2022).

The preferential accumulation of these locus-specific methylation changes within transposable elements, accounting for 97-100% of CHG-DMRs and 51-55% of CHH-DMRs in both genotypes, suggests that TE-associated chromatin remodeling is a central feature of the Al stress epigenetic response in rice. TE methylation changes under stress have been associated with the mobilization of regulatory elements that influence nearby gene expression, potentially contributing to adaptive transcriptional reprogramming (Hirsch and Springer, 2017; Lisch, 2013). The stronger TE-associated CHH hypomethylation observed in BGI relative to Azucena is consistent with a more extensive relaxation of TE silencing in the susceptible genotype, which may contribute to the broader and less specific transcriptional response observed in BGI.

### 4.3 DNA methylation and transcriptional changes reflect contrasting Al adaptation strategies in tolerant and susceptible rice genotypes

Al toxicity imposes severe constraints on root growth and nutrient acquisition in acidic soils, and plant tolerance depends on the rapid and coordinated activation of specialized detoxification pathways. The comparative methylome-transcriptome analysis presented in this study revealed fundamentally different epigenetic strategies for coping with Al stress between susceptible and tolerant genotypes. Although both genotypes activate stress-associated genes and share a common set of methylation-responsive loci, the nature, specificity, and regulatory coherence of their epigenetic responses diverge sharply, providing mechanistic insights into their contrasting phenotypes. We acknowledge that the stringent |log_2_FC| ≥ 2 threshold applied to identify DEGs for methylation-expression integration may have excluded genes with smaller but biologically relevant expression changes. However, genes exhibiting higher fold-changes are generally detected more robustly and reproducibly across biological replicates (Bourdon-Lacombe et al., 2015). Therefore, the use of a stringent threshold allowed us to focus on high-confidence transcriptional responses that were more likely to represent biologically meaningful regulatory events associated with differential DNA methylation.

Among the genes that were both differentially methylated and expressed, we only found three genes shared between both genotypes: *CCD8A* (Os01g0566500), *UGT93B9* (Os04g0556400), and *FDH2* (Os06g0486900), further underscoring the genotype-dependent nature of the methylation-expression relationship. Remarkably, all three genes showed opposite transcriptional responses between genotypes despite overlapping methylation changes: repression in Azucena and activation in BGI in all cases. CCD8A encodes a dioxygenase involved in carlactone and strigolactone biosynthesis, phytohormones with emerging roles in regulating root architecture and abiotic stress adaptation (Kapulnik and Koltai, 2014). *UGT93B9* encodes a UDP-glycosyltransferase involved in cytokinin glycosylation, which modulates cytokinin bioavailability and homeostasis. Cytokinin glycosyltransferases have been directly implicated in abiotic stress responses in rice (Dong et al., 2020; Kudo et al., 2012). Finally, FDH2 encodes a mitochondrial formate dehydrogenase, the expression of which is induced in root tips by Al stress, where FDH confers increased Al and H^+^ tolerance by reducing stress-induced formate accumulation in root cells (Lou et al., 2016). The repression of these genes in Azucena and activation in BGI under identical upstream CHH hypomethylation may reflect differences in the downstream transcriptional regulatory context between genotypes, suggesting that the same epigenetic signal can produce divergent transcriptional outcomes depending on the chromatin and regulatory environment of the loci.

To understand the different mechanisms that can occur for each genotype under Al stress, we identified differentially methylated and expressed genes specific to each genotype that were directly associated with stress responses. In BGI, the hypomethylation-driven transcriptional response to Al stress is dominated by the induction of broad stress-responsive gene families rather than Al-specific detoxification mechanisms. Upregulated genes with coordinated upstream hypomethylation included dioxygenases (Os01g0566500), UDP-glycosyltransferases (Os07g0502900, Os05g0526900, Os05g0527100), oxo-phytodienoate reductases (Os06g0215600), lipases (Os11g0521000, Os01g0216000), cytochrome P450s (Os10g0167200, Os09g0530300), and SAUR auxin-responsive proteins (Os12g0626200). These gene families participate in broad abiotic and biotic stress responses, integrating signals from drought, salinity, cold, oxidative stress, pathogens, and hormone fluctuations, but are not specifically associated with Al detoxification. UDP-glycosyltransferases adjust hormone signaling strength, ROS balance, and detoxification capacity in response to various stressors (Li et al., 2017; Ma et al., 2025; Rehman et al., 2018). Oxo-phytodienoate reductases modulate ABA-dependent signaling and antioxidant pathways under multiple stresses (Dong et al., 2013; Mou et al., 2025), whereas plant lipases remodel membranes and generate lipid messengers that help plants survive environmental challenges (Ali et al., 2022; Sagar and Singh, 2021). Cytochrome P450s broadly contribute to hormone balance, antioxidant defense, and specialized metabolite production under stress (Chakraborty et al., 2023; Pandian et al., 2020), and SAUR proteins modulate ABA signaling and root growth responses to abiotic stress (Guo et al., 2018; He et al., 2021). Complementarily, key genes were upregulated when hypomethylation was detected across gene bodies. MYB transcription factors are known to participate in stress responses and can be modulated by DNA methylation (Liu et al., 2019), aligning with our finding of differentially methylated and expressed MYB genes in BGI, Os08g0437300 and Os07g0558100 are members of the MYB family that were triggered when Al was experimentally induced.

BGI plants also upregulated genes scattered through mixed molecular functions associated with various biochemical alternatives to cope with Al stress. Genes that code for AMP and NAD(P)-binding proteins (Os03g0305100 and Os10g0576900, respectively), calcium signaling (Os03g0310800), vitamin B6 biosynthesis (Os07g0100200), and chloroplast enzymes (Os10g0496900) were highly expressed upon promoter hypomethylation. These patterns might indicate that BGI displays diffuse, nonspecific stress responses rather than a targeted Al detoxification program. While this generalized activation might reflect an attempt to mitigate cellular damage, it lacks the mechanistic precision required to neutralize Al³^+^ toxicity at the root-soil interface.

In addition to this nonspecific activation, BGI also displays hypermethylation and transcriptional repression of key regulatory genes essential for coordinating abiotic stress responses. Notably, the AP2/ERF transcription factor Os10g0562900, a major hub in plant stress signaling that orchestrates gene networks involved in drought, salinity, cold, heavy metal stress, and hormone regulation (Ma et al., 2024; Wang et al., 2024; Xie et al., 2019), was hypermethylated and downregulated in BGI. Similarly, hypermethylation and reduced expression of the UDP-glycosyltransferase Os07g0502900 further limits the activity of a gene family that modulates hormone homeostasis, antioxidant capacity, and detoxification. These epigenetic constraints suggest that the BGI stress response is not only nonspecific but also regulatorily impaired, preventing the establishment of a coordinated and effective adaptive program against Al toxicity (**Figure 5**).

In contrast, Azucena displayed a more targeted and mechanistically coherent epigenetic response to Al stress, with coordinated methylation and expression changes concentrated in genes directly relevant to known Al tolerance mechanisms. One of the most interesting examples is Os05g0219900, a C4-dicarboxylate/malic acid transporter, whose upstream hypomethylation correlates with transcriptional activation in Azucena. Malate efflux is one of the best-characterized mechanisms of Al exclusion in plants, operating through ALMT-type transporters that chelate Al³^+^ in the rhizosphere (Kobayashi et al., 2013). The epigenetic activation of a malate transport gene, specifically in the tolerant genotype, provides a mechanistic basis for understanding how Azucena maintains effective Al exclusion under prolonged stress. In addition, Azucena showed coordinated methylation changes in genes associated with the MATE family transporters (Os10g0345100, Os08g0480000), the same protein family that includes OsFRDL4, the major citrate transporter responsible for Al exclusion in rice roots (Gallo-Franco et al., 2023; Yokosho et al., 2011), further reinforcing the link between epigenetic regulation and organic acid-mediated Al detoxification in the tolerant genotype.

Azucena also activates aquaporins (Os03g0861300), which regulate water flow and membrane stability under metal toxicity (Afzal et al., 2016; Gautam and Pandey, 2021), and flavin-dependent monooxygenases (Os03g0182000), which regulate ROS homeostasis and auxin and ABA pathways under stress (Gaba et al., 2023; L. Wang et al., 2023). Additionally, the probenazole-inducible protein PBZ1 (Os12g0555100), which is strongly induced by multiple abiotic stresses and capable of triggering programmed cell death as a defense-related response (Kim et al., 2008; Tanaka et al., 2006), showed upstream CHH hypomethylation and transcriptional activation exclusively in Azucena, suggesting that epigenetic regulation of stress-induced cell death pathways may also contribute to the tolerant phenotype. Together, these genes structure a coordinated network that integrates Al exclusion, ROS control, membrane stability, and cellular protection, reinforcing our hypothesis of a precise epigenetic regulation in shaping stress resilience in the tolerant genotype.

A key consideration for interpreting these results is that the methylome and transcriptome data were collected after 10 days of continuous Al exposure, a timeframe that, particularly for the tolerant genotype Azucena, likely represents an adaptive rather than an acute stress state. Under this interpretation, the gene repression observed in Azucena may not reflect the active silencing of stress-response pathways but rather the return to a regulated baseline following an earlier phase of activation, consistent with a successful adaptive response. In contrast, the persistent broad activation observed in BGI may reflect an inability to resolve the stress response and establish a stable adaptive program even after prolonged exposure. It should also be noted that Azucena and BGI were both mapped to the reference genome of Nipponbare rice genotype rather than to genotype-specific assemblies. Since neither variety is genetically identical to Nipponbare, some of the genotype-specific methylation and expression patterns discussed above should be interpreted with this shared reference constraint in mind.

Overall, the contrasting epigenetic responses of BGI and Azucena illustrate two fundamentally different strategies: BGI relies on a broad, nonspecific, and partially regulatory-impaired stress program driven by diffuse epigenomic perturbation, whereas Azucena orchestrates a precise, epigenetically enabled Al-tolerance mechanism centered on organic acid transport, ROS regulation, and membrane stability. These findings underscore the importance of epigenetic regulatory specificity, rather than just the magnitude of methylation changes, as a determinant of Al tolerance in rice and highlight candidate epigenetically regulated genes and pathways for breeding or engineering more resilient cultivars.

## 5 Conclusions

This study provides the first genome-wide characterization of DNA methylation dynamics and their transcriptional consequences under Al stress in rice, suggesting that epigenomic reprogramming under Al exposure is sequence-context-specific and genotype-dependent.

CHG methylation was broadly reduced under stress in both genotypes, with a significantly stronger response in BGI, while CHH methylation showed a genome-wide increase in BGI but more subtle changes in Azucena, suggesting more extensive epigenomic perturbation in the susceptible background. The global CHH hypermethylation observed in BGI may reflect an active genome defense response mediated by the RdDM pathway aimed at reinforcing TE silencing under stress or may serve as a direct DNA-protective function against ROS-induced strand breaks that have been proposed for Al toxicity in root cells. Finally, the concentration of DMRs within transposable elements, particularly in the CHG and CHH contexts, identifies TE-associated chromatin remodeling as a central feature of the Al stress epigenetic response, whereas the enrichment of terpenoid biosynthesis pathways among DMR-associated genes points to the epigenetic regulation of secondary metabolism as a shared component of the Al stress response across genotypes.

At the level of methylation-expression changes, the two genotypes shared only three differentially methylated and expressed genes, all showing opposite transcriptional responses among genotypes, underscoring the functional dependence on the genetic background. The gathered evidence suggests that Azucena displayed a coherent, epigenetically enabled Al-tolerance program centered on organic acid transporters, MATE family members, aquaporins, and ROS-regulatory enzymes, whereas BGI showed a broader, less specific response characterized by the activation of general stress-responsive genes and repression of key transcriptional regulators (**Figure 5**).

Together, these findings establish DNA methylation as an additional regulatory layer shaping Al tolerance in rice, with the candidate genes identified here representing promising targets for epigenome-assisted breeding. Future studies integrating time-course analyses, single-cell epigenomics, and functional validation of key candidates are essential to resolve the causal relationships between methylation dynamics and adaptive transcriptional reprogramming under Al stress.

## Data Availability

The raw WGBS data analyzed in this paper can be found in the NCBI Sequence Read Archive (SRA) database under BioProject accession number PRJNA1478175. The raw RNA sequencing data can be found in the NCBI SRA database under BioProject accession number PRJNA980182. Datasets and scripts used for these analyses are available in the GitHub repository: https://github.com/ccsosa/Rice_aluminum_stress_methylated_transcriptome.
