# Supplementary material for: Integrative methylome and transcriptome analysis reveals genotype and sequence context-specific responses to aluminum stress in rice
Source: Front Plant Sci. 2026 Jul 31;17:1896320. doi: 10.3389/fpls.2026.1896320 (PMC13474991; doi:10.3389/fpls.2026.1896320)
Supplement: Supplementary file 1 [file DataSheet1.pdf]

Annex 1.

RNA extraction protocol from roots and leaves for complex samples.

1. Add 1mL of Trizol to 200mg of tissue macerated in liquid nitrogen. 2.
2. Vortex for 5 min.
3. Incubate for 2 hours at 4°C and occasionally shake manually.
4. Centrifuge at 14000 rpm for 10min at 4°C.
5. Transfer 900uL of the supernatant to a new tube.
6. Add 500uL of PCI and vortex for 2min.
7. Incubate at 4°C for 10min with manual shaking every 2.5min.
8. Centrifuge at 14000rpm for 10 min at 4°C.
9. Transfer 400uL of the supernatant to a new tube.
10. Add 400uL of chloroform and vortex 30s.
11. Incubate for 3min at 4°C.
12. Centrifuge at 14000rpm for 15 min at 4°C.
13. Transfer the supernatant (approx. 300uL) to a new tube.
14. Add 300uL of chloroform and mix in vortex for 30s.
15. Incubate for 3min at 4°C.
16. Centrifuge at 14000rpm for 10 min at 4°C.
17. Transfer the supernatant (approx. 250uL) to a new tube.
18. Add 500uL of isopropanol and 80uL of 10M LiCl (should be at a final concentration of approximately 1M).
19. Incubate over night at -20°C.
20. Centrifuge at 14000rpm for 15min at 4°C.
21. Discard supernatant.
22. Add 1mL of 75% ethanol and vortex very briefly.
23. Centrifuge at 14000rpm for 10min at 4°C.
24. Discard the supernatant.
25. Add 500uL of 75% ethanol. 26.
26. Centrifuge at 14000rpm for 10min at 4°C.
27. Discard the supernatant
28. Allow the pellet to dry
29. Resuspend the pellet in 50uL of inactivated DEPC water.
