# Supplementary material for: Integrative methylome and transcriptome analysis reveals genotype and sequence context-specific responses to aluminum stress in rice
Source: Front Plant Sci. 2026 Jul 31;17:1896320. doi: 10.3389/fpls.2026.1896320 (PMC13474991; doi:10.3389/fpls.2026.1896320)
Supplement: Supplementary file 2 [file Image2.pdf]

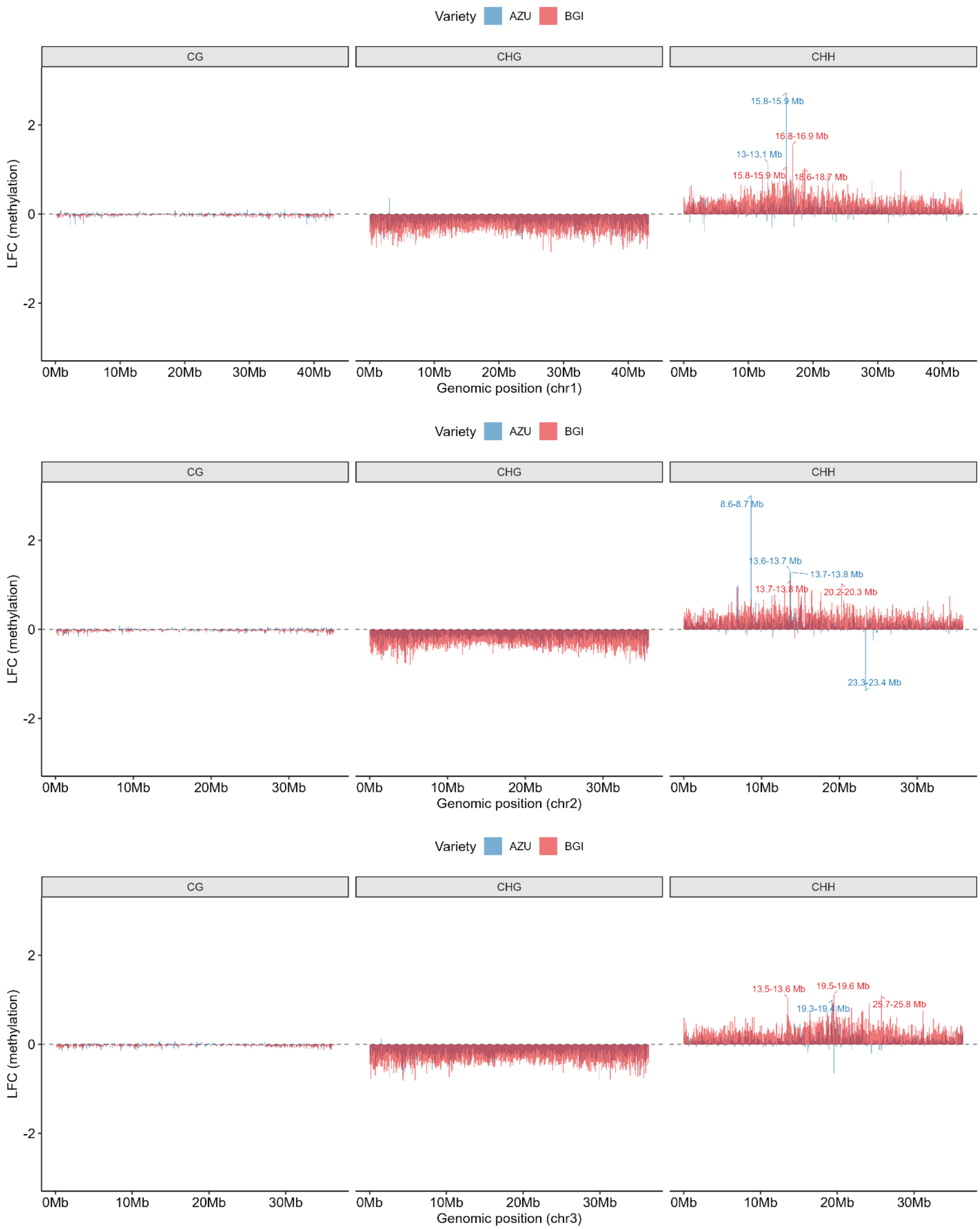
Variety AZU BGI
CG
CHG
CHH
LFC (methylation)
2
0
-2
15.8-15.9 Mb
16.8-16.9 Mb
13-13.1 Mb
15.8-15.9 Mb
18.6-18.7 Mb
0Mb 10Mb 20Mb 30Mb 40Mb
Genomic position (chr1)
8.6-8.7 Mb
13.6-13.7 Mb
13.7-13.8 Mb
13.7-13.8 Mb
20.2-20.3 Mb
23.3-23.4 Mb
0Mb 10Mb 20Mb 30Mb
Genomic position (chr2)
13.5-13.6 Mb
19.5-19.6 Mb
19.3-19.4 Mb
25.7-25.8 Mb
Genomic position (chr3)

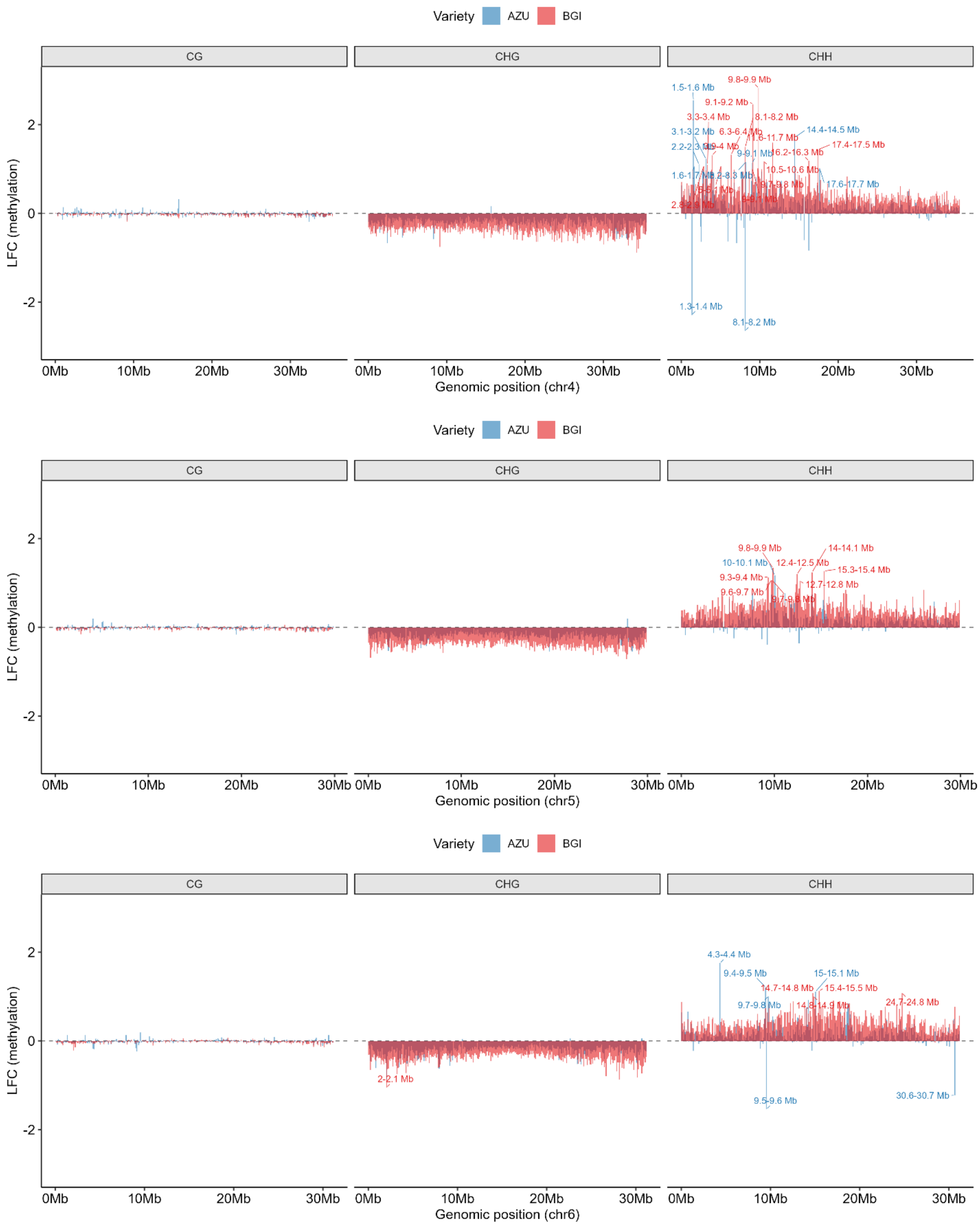

Variety
AZU
BGI
CG
CHG
CHH
LFC (methylation)
2
0
-2
0Mb
10Mb
20Mb
30Mb
Genomic position (chr4)
Variety
AZU
BGI
CG
CHG
CHH
LFC (methylation)
Genomic position (chr5)
Variety
AZU
BGI
CG
CHG
CHH
LFC (methylation)
Genomic position (chr6)

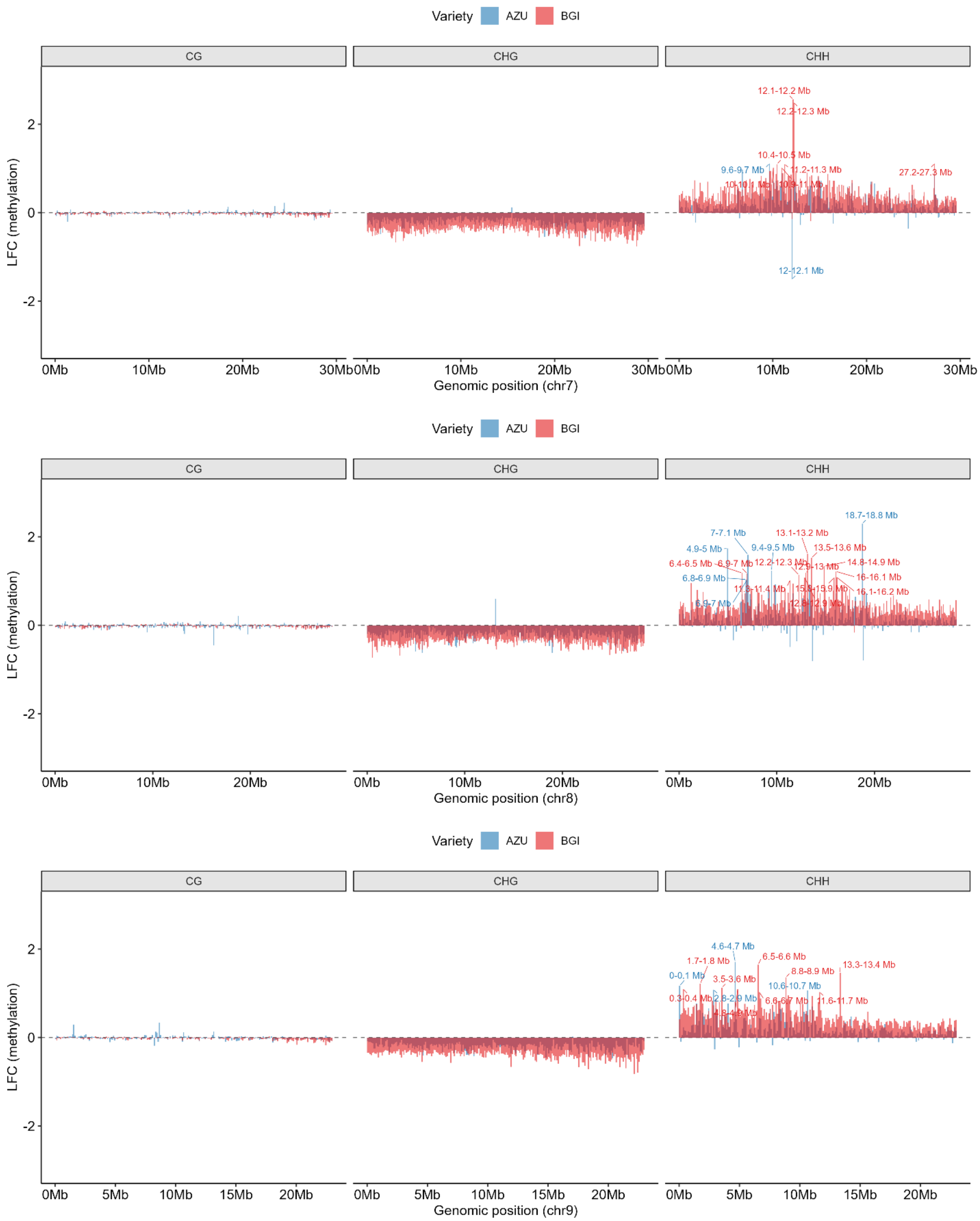

Variety AZU BGI
CG
CHG
CHH
LFC (methylation)
2
0
-2
0Mb
10Mb
20Mb
30Mb
Genomic position (chr7)
12.1-12.2 Mb
12.2-12.3 Mb
10.4-10.5 Mb
9.6-9.7 Mb
11.2-11.3 Mb
27.2-27.3 Mb
12-12.1 Mb
Genomic position (chr8)
18.7-18.8 Mb
7-7.1 Mb
13.1-13.2 Mb
4.9-5 Mb
9.4-9.5 Mb
13.5-13.6 Mb
6.4-6.5 Mb
12.2-12.3 Mb
14.8-14.9 Mb
6.8-6.9 Mb
16-16.1 Mb
16.1-16.2 Mb
Genomic position (chr9)
5Mb
15Mb
4.6-4.7 Mb
1.7-1.8 Mb
6.5-6.6 Mb
13.3-13.4 Mb
0-0.1 Mb
3.5-3.6 Mb
8.8-8.9 Mb
10.6-10.7 Mb
11.6-11.7 Mb

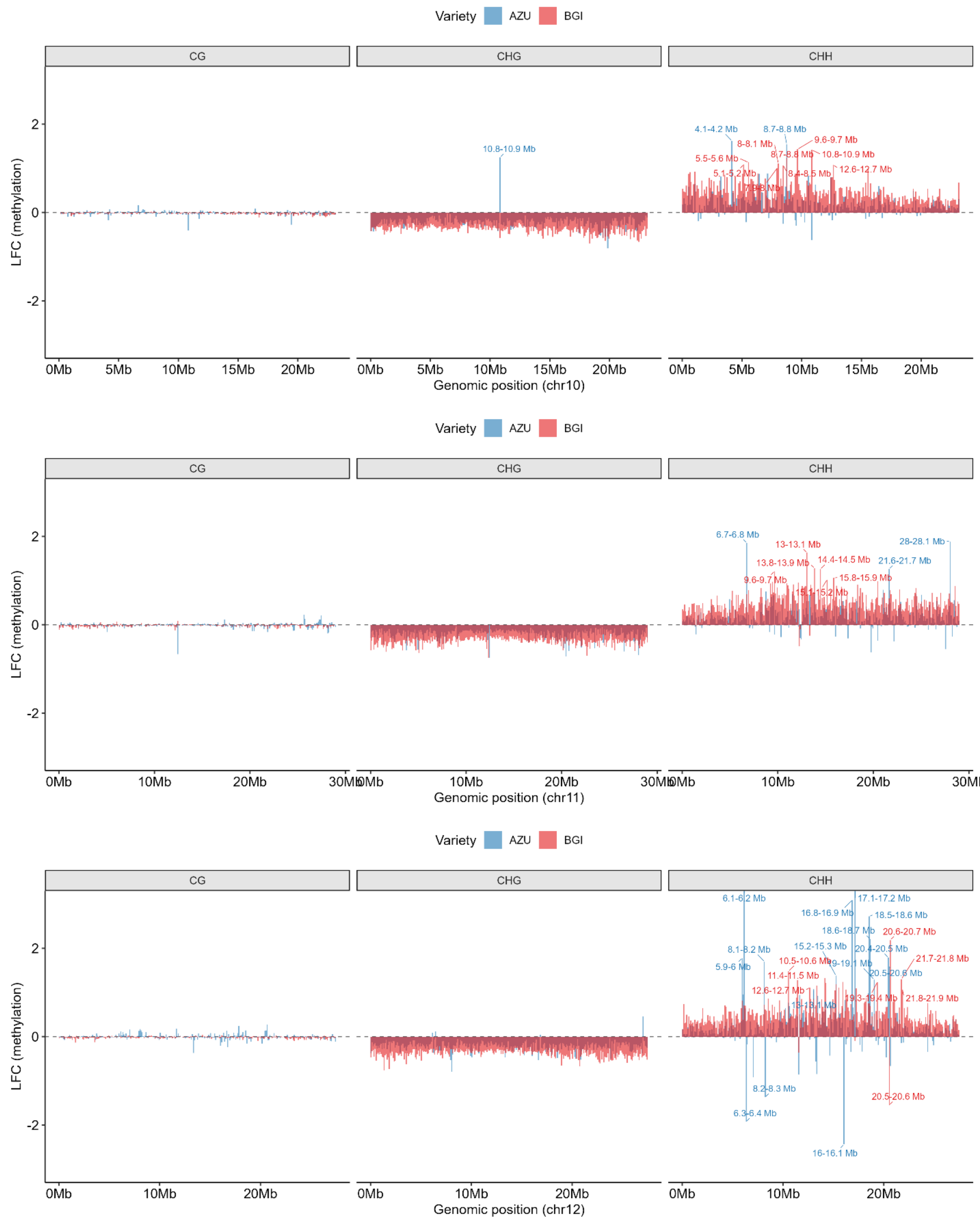

Supplementary Figure S2. Genome-wide distribution of the log2 fold change (LFC) in methylated cytosine (mC) counts across 100-kb genomic windows under aluminum (Al) stress, for the CG, CHG, and CHH contexts, in Azucena (Al-tolerant) and BGI (BGI, Al-sensitive) rice genotypes, shown separately for each of the 12 rice chromosomes. Each line represent the log2FC in mC counts (Al stress relative to control) per 100-kb window.
