# Supplementary material for: Integrative methylome and transcriptome analysis reveals genotype and sequence context-specific responses to aluminum stress in rice
Source: Front Plant Sci. 2026 Jul 31;17:1896320. doi: 10.3389/fpls.2026.1896320 (PMC13474991; doi:10.3389/fpls.2026.1896320)
Supplement: Supplementary file 3 [file Image3.pdf]

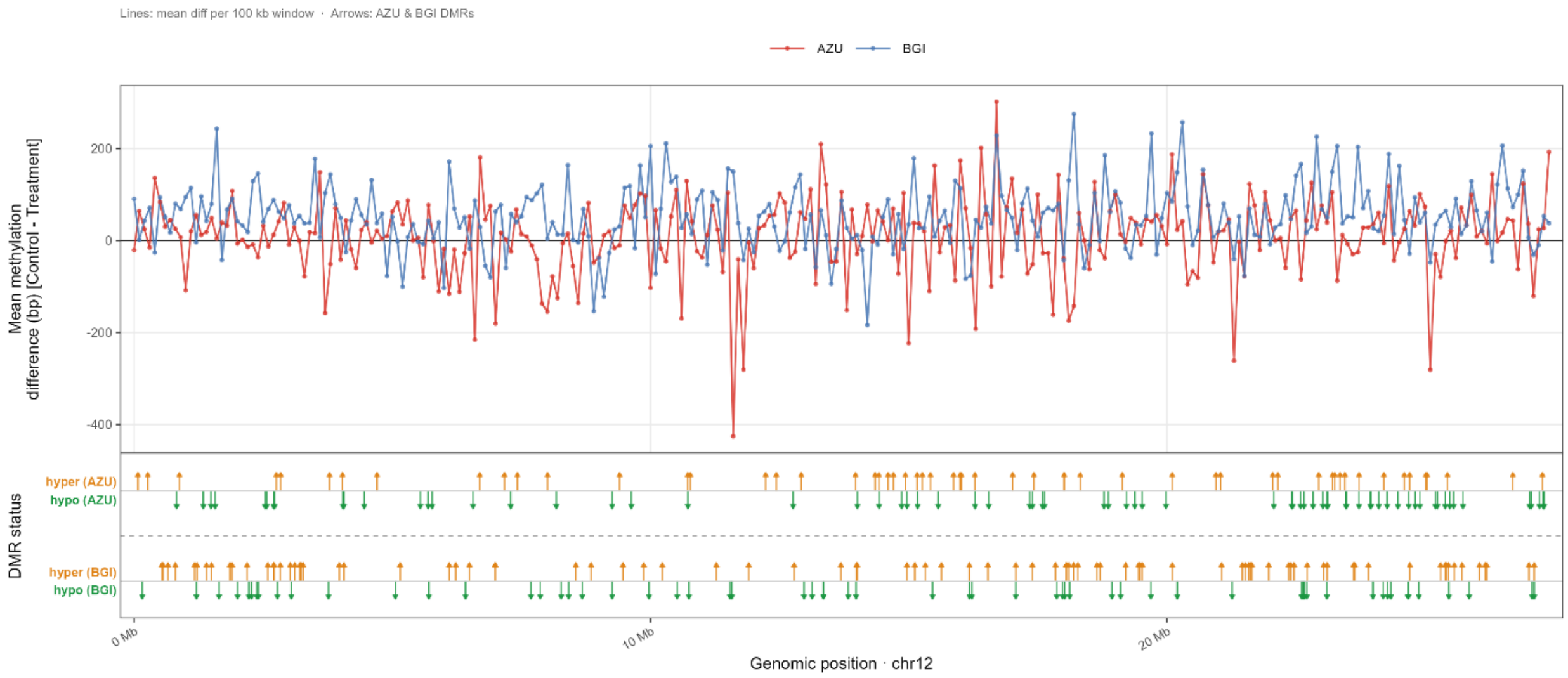
Lines: mean diff per 100 kb window · Arrows: AZU & BGI DMRs
AZU
BGI
Mean methylation difference (bp) [Control - Treatment]
200
0
-200
-400
DMR status
hyper (AZU)
hypo (AZU)
hyper (BGI)
hypo (BGI)
0 Mb
10 Mb
20 Mb
Genomic position · chr12

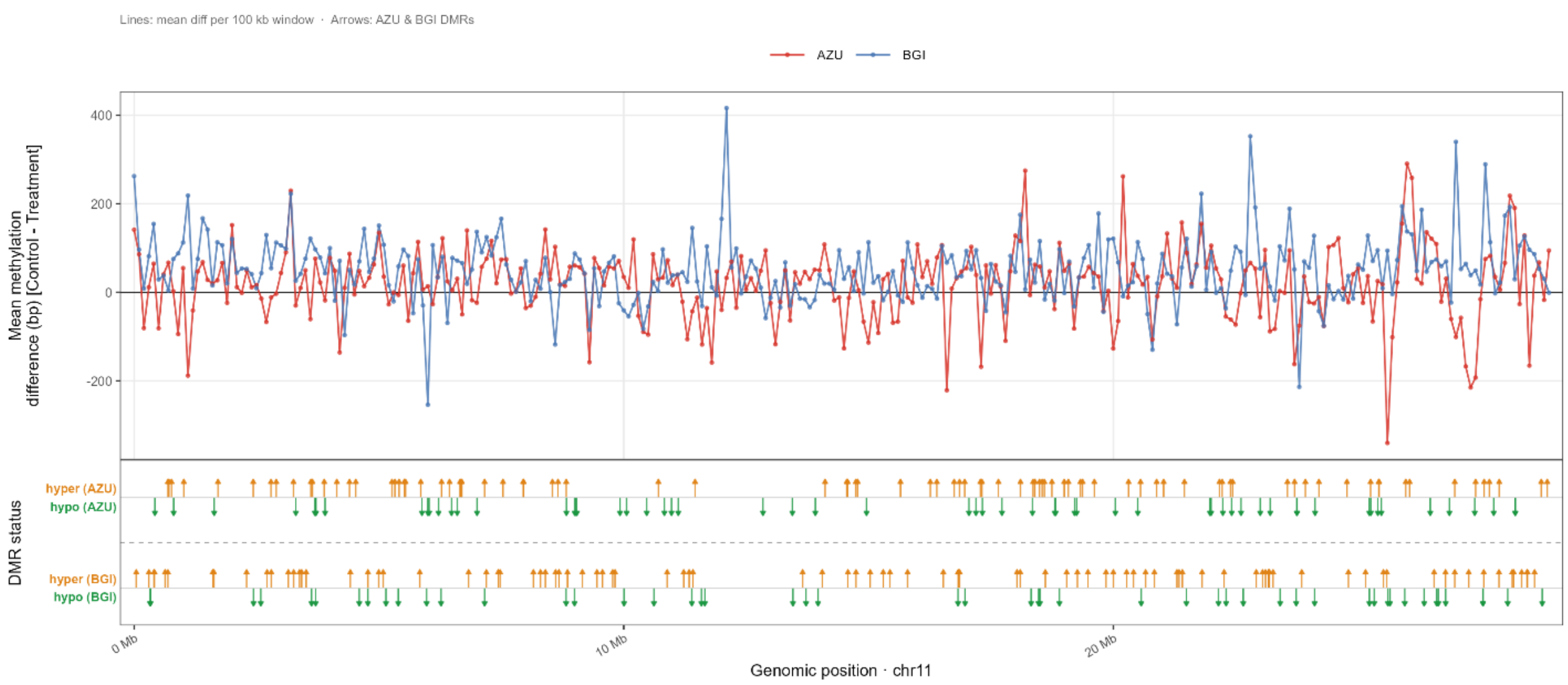
Lines: mean diff per 100 kb window · Arrows: AZU & BGI DMRs
AZU
BGI
Mean methylation difference (bp) [Control - Treatment]
400
200
0
-200
DMR status
hyper (AZU)
hypo (AZU)
hyper (BGI)
hypo (BGI)
0 Mb
10 Mb
20 Mb
Genomic position · chr11

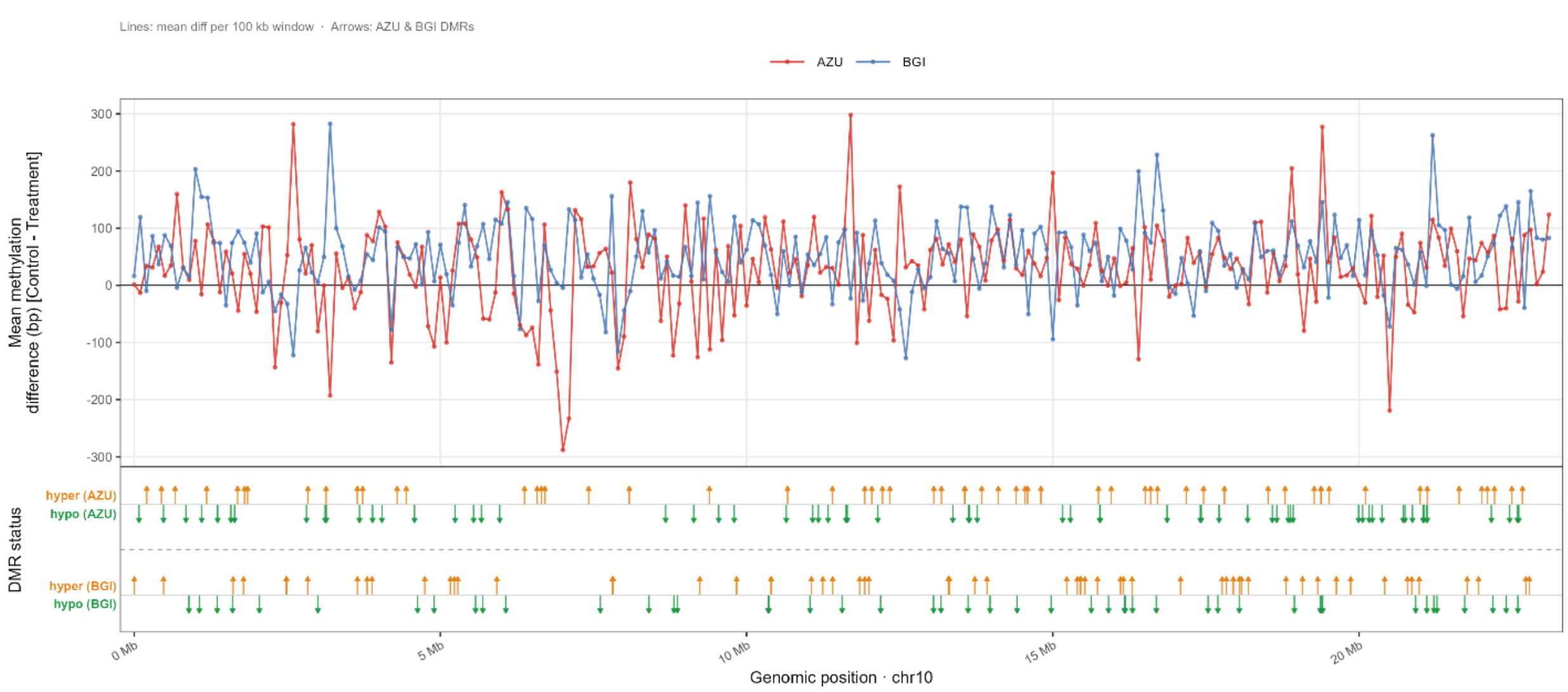
Lines: mean diff per 100 kb window · Arrows: AZU & BGI DMRs
AZU
BGI
Mean methylation difference (bp) [Control - Treatment]
300
200
100
0
-100
-200
-300
DMR status
hyper (AZU)
hypo (AZU)
hyper (BGI)
hypo (BGI)
0 Mb
5 Mb
10 Mb
15 Mb
20 Mb
Genomic position · chr10

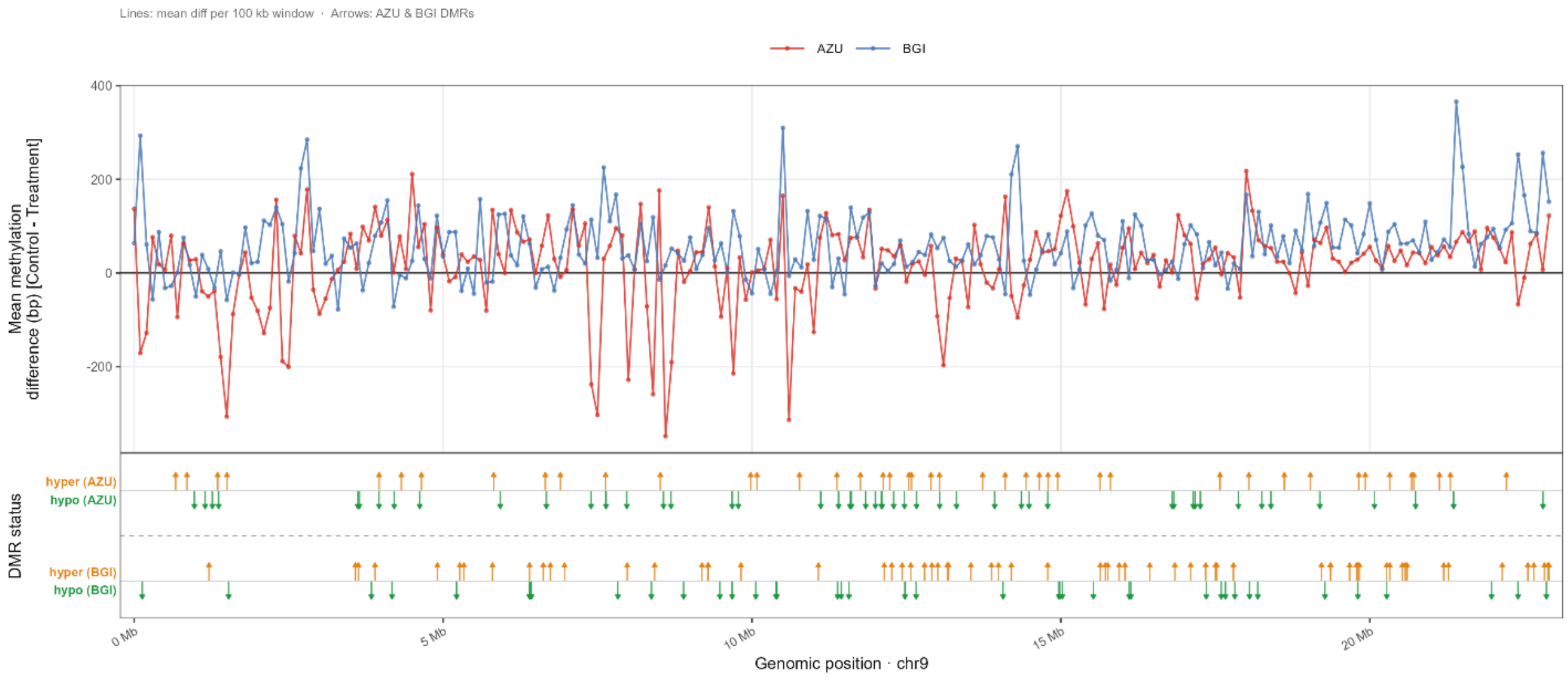
Lines: mean diff per 100 kb window · Arrows: AZU & BGI DMRs
AZU
BGI
400
200
0
-200
Mean methylation difference (bp) [Control - Treatment]
hyper (AZU)
hypo (AZU)
hyper (BGI)
hypo (BGI)
DMR status
0 Mb
5 Mb
10 Mb
15 Mb
20 Mb
Genomic position · chr9

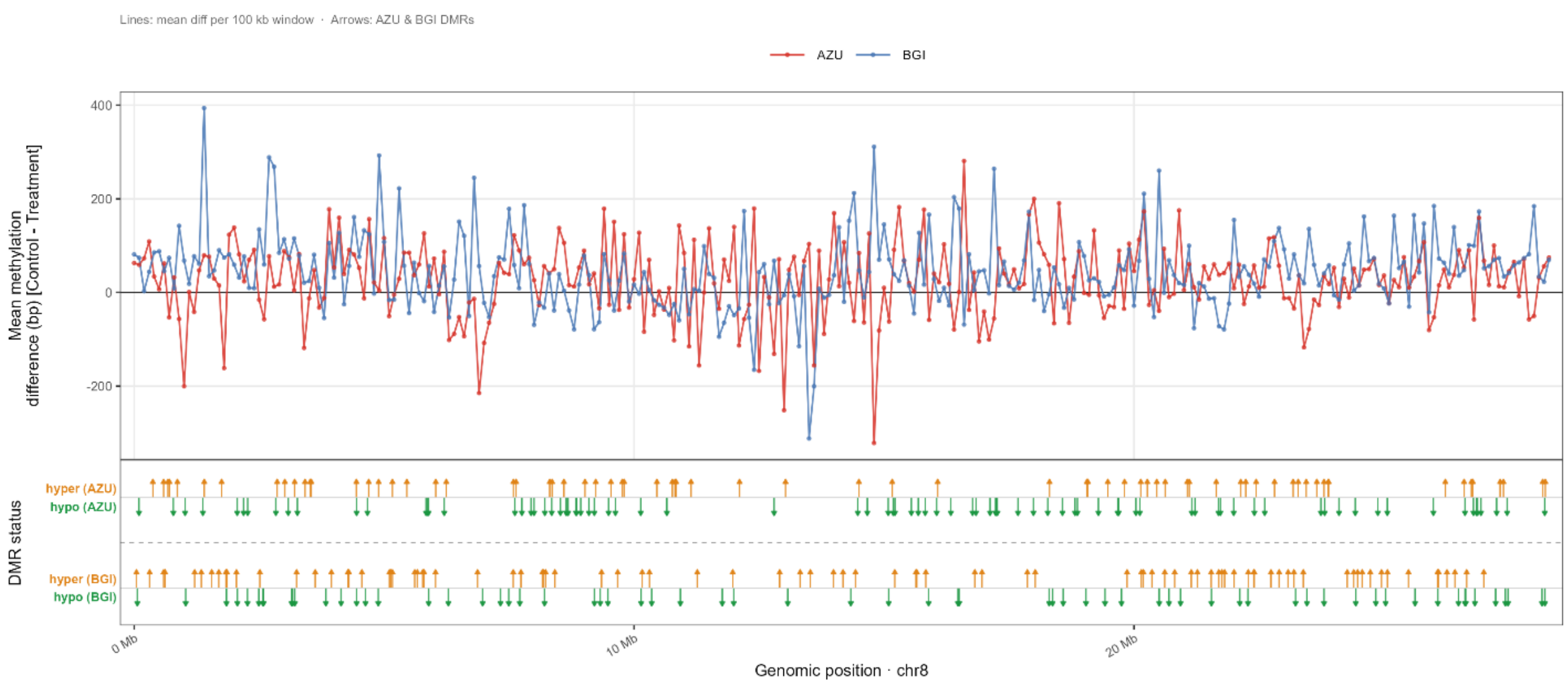
Lines: mean diff per 100 kb window · Arrows: AZU & BGI DMRs
AZU
BGI
400
200
0
-200
Mean methylation difference (bp) [Control - Treatment]
hyper (AZU)
hypo (AZU)
hyper (BGI)
hypo (BGI)
DMR status
0 Mb
10 Mb
20 Mb
Genomic position · chr8

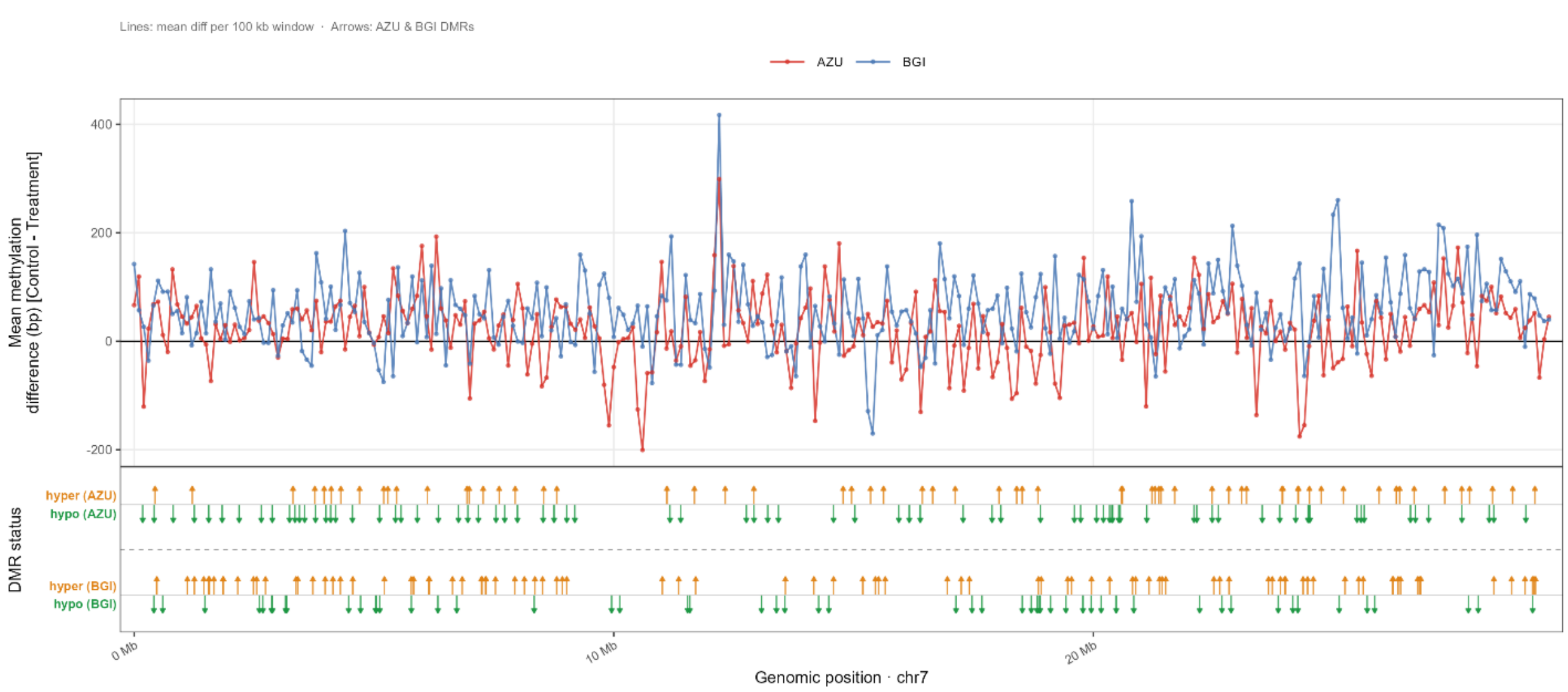
Lines: mean diff per 100 kb window · Arrows: AZU & BGI DMRs
AZU
BGI
400
200
0
-200
Mean methylation difference (bp) [Control - Treatment]
hyper (AZU)
hypo (AZU)
hyper (BGI)
hypo (BGI)
DMR status
0 Mb
10 Mb
20 Mb
Genomic position · chr7

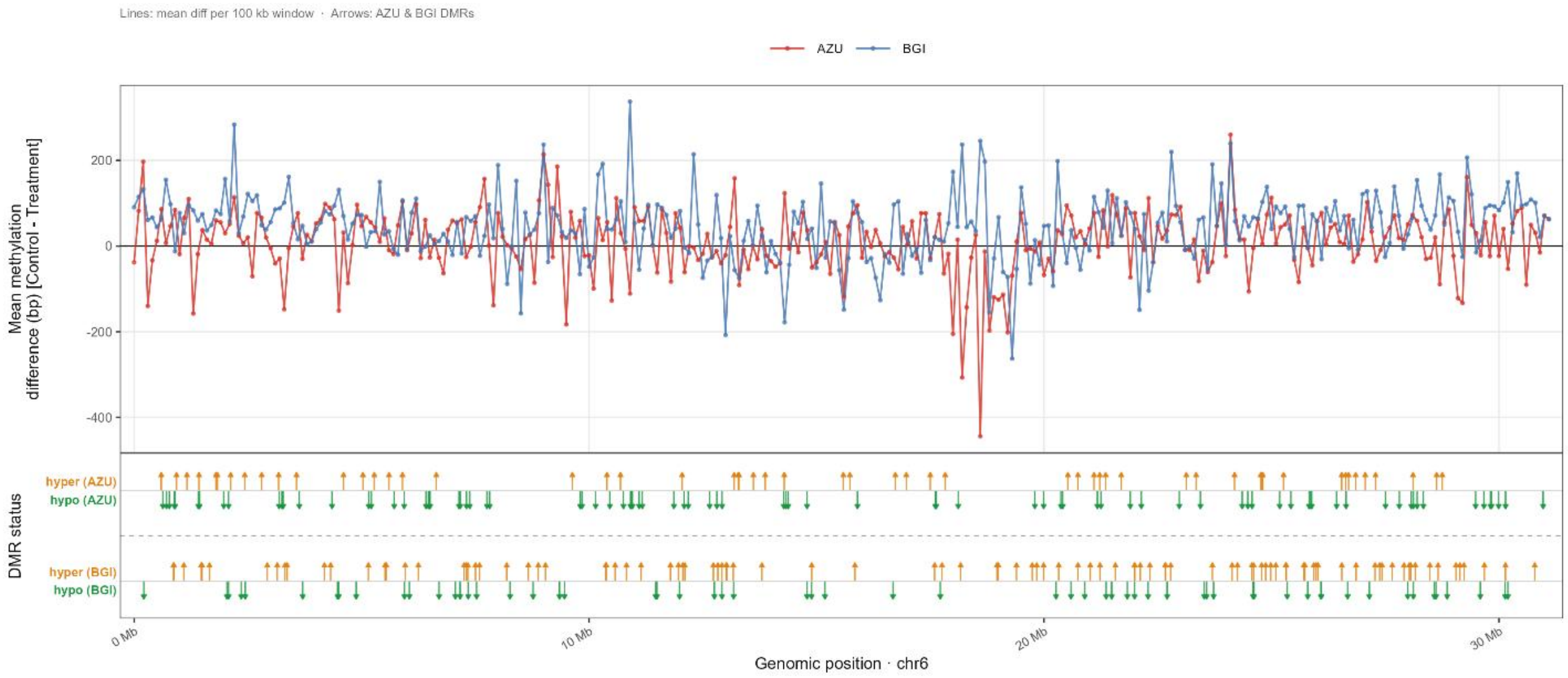
Lines: mean diff per 100 kb window · Arrows: AZU & BGI DMRs
AZU
BGI
Mean methylation difference (bp) [Control - Treatment]
200
0
-200
-400
DMR status
hyper (AZU)
hypo (AZU)
hyper (BGI)
hypo (BGI)
0 Mb
10 Mb
20 Mb
30 Mb
Genomic position · chr6

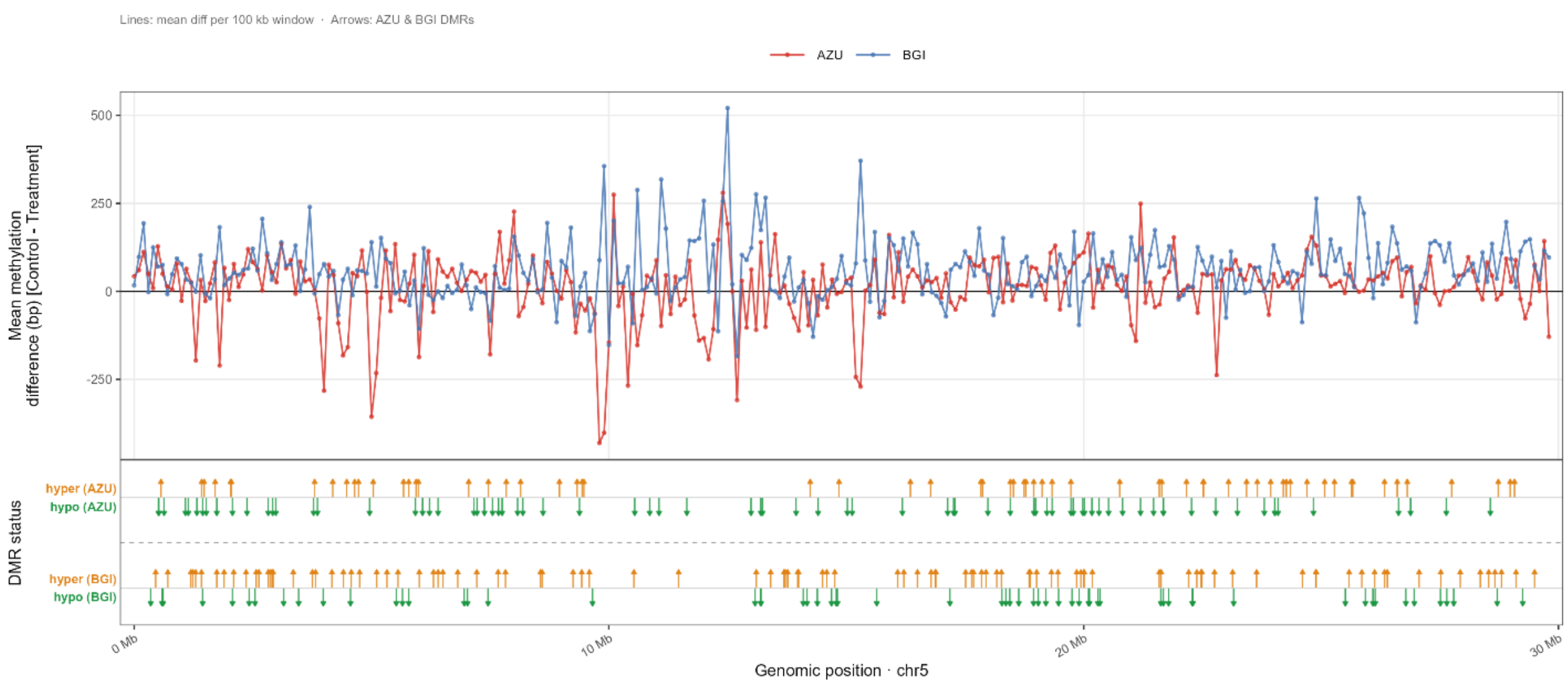
Lines: mean diff per 100 kb window · Arrows: AZU & BGI DMRs
AZU
BGI
Mean methylation difference (bp) [Control - Treatment]
500
250
0
-250
DMR status
hyper (AZU)
hypo (AZU)
hyper (BGI)
hypo (BGI)
0 Mb
10 Mb
20 Mb
30 Mb
Genomic position · chr5

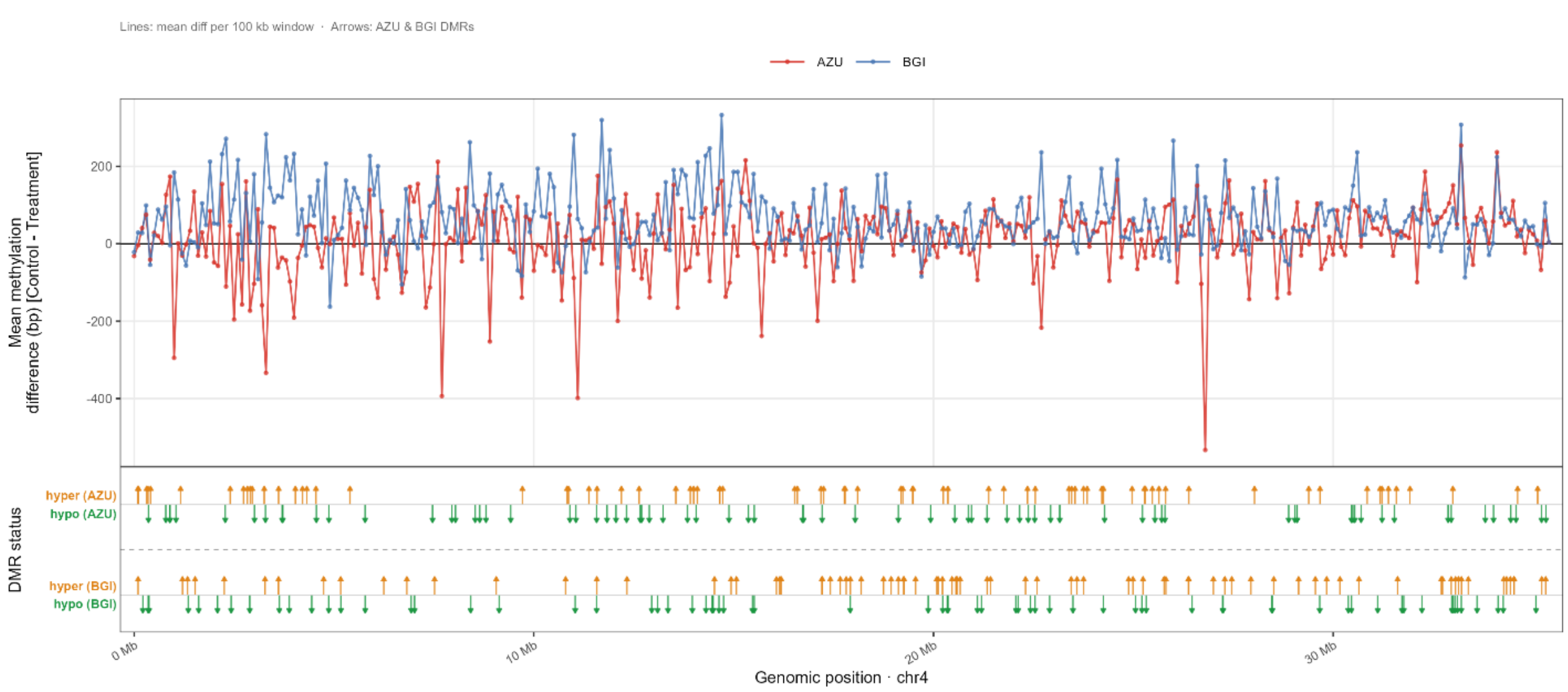
Lines: mean diff per 100 kb window · Arrows: AZU & BGI DMRs
AZU
BGI
Mean methylation difference (bp) [Control - Treatment]
200
0
-200
-400
DMR status
hyper (AZU)
hypo (AZU)
hyper (BGI)
hypo (BGI)
0 Mb
10 Mb
20 Mb
30 Mb
Genomic position · chr4

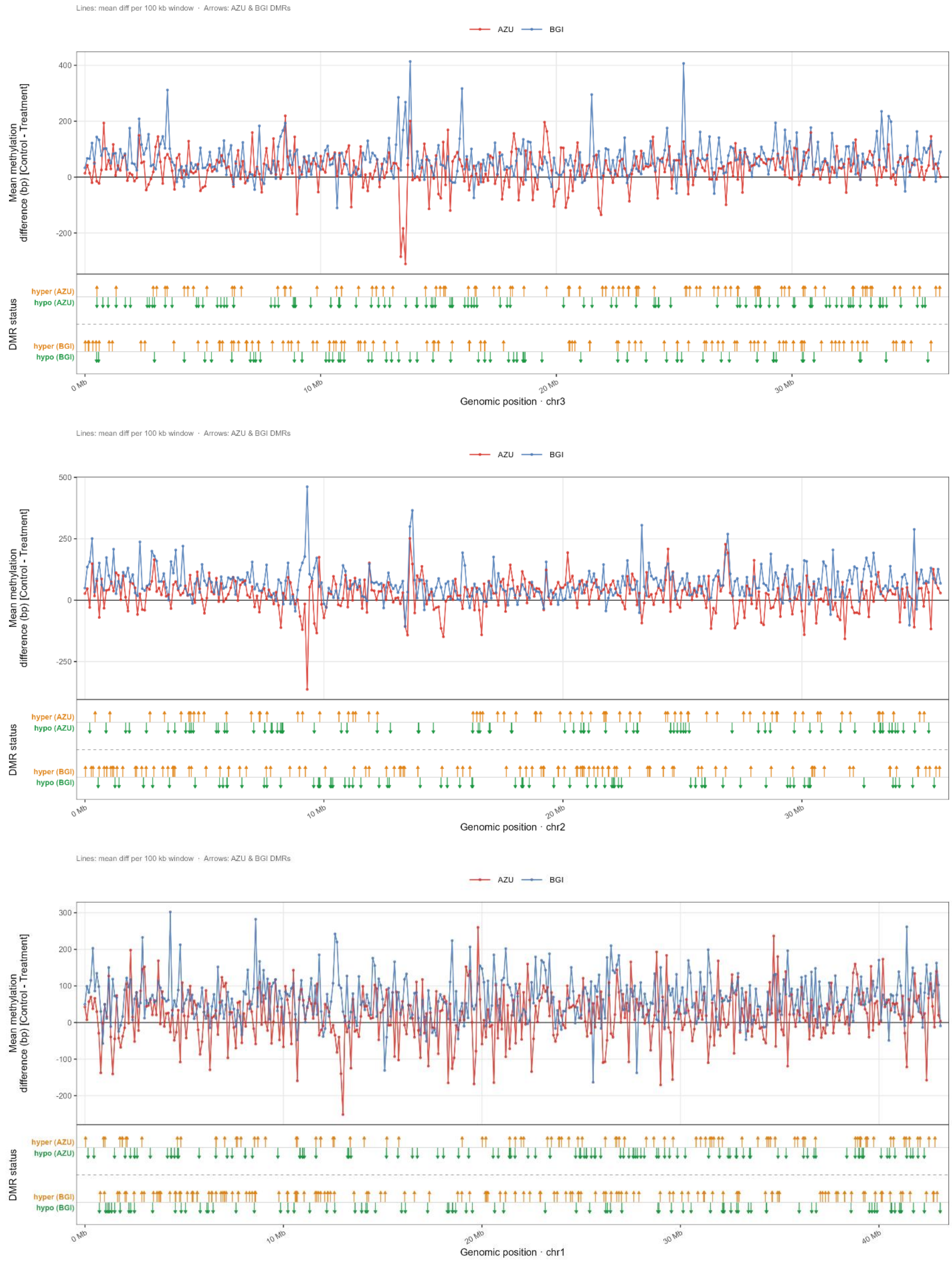

Supplementary Figure S3. Genome-wide distribution of DNA methylation differences at the CG context between control and stress conditions, and differentially methylated regions (DMRs) across chromosomes in AZU and BGI rice genotypes under aluminum stress.
