# Supplementary material for: Integrative methylome and transcriptome analysis reveals genotype and sequence context-specific responses to aluminum stress in rice
Source: Front Plant Sci. 2026 Jul 31;17:1896320. doi: 10.3389/fpls.2026.1896320 (PMC13474991; doi:10.3389/fpls.2026.1896320)
Supplement: Supplementary file 4 [file Image4.pdf]

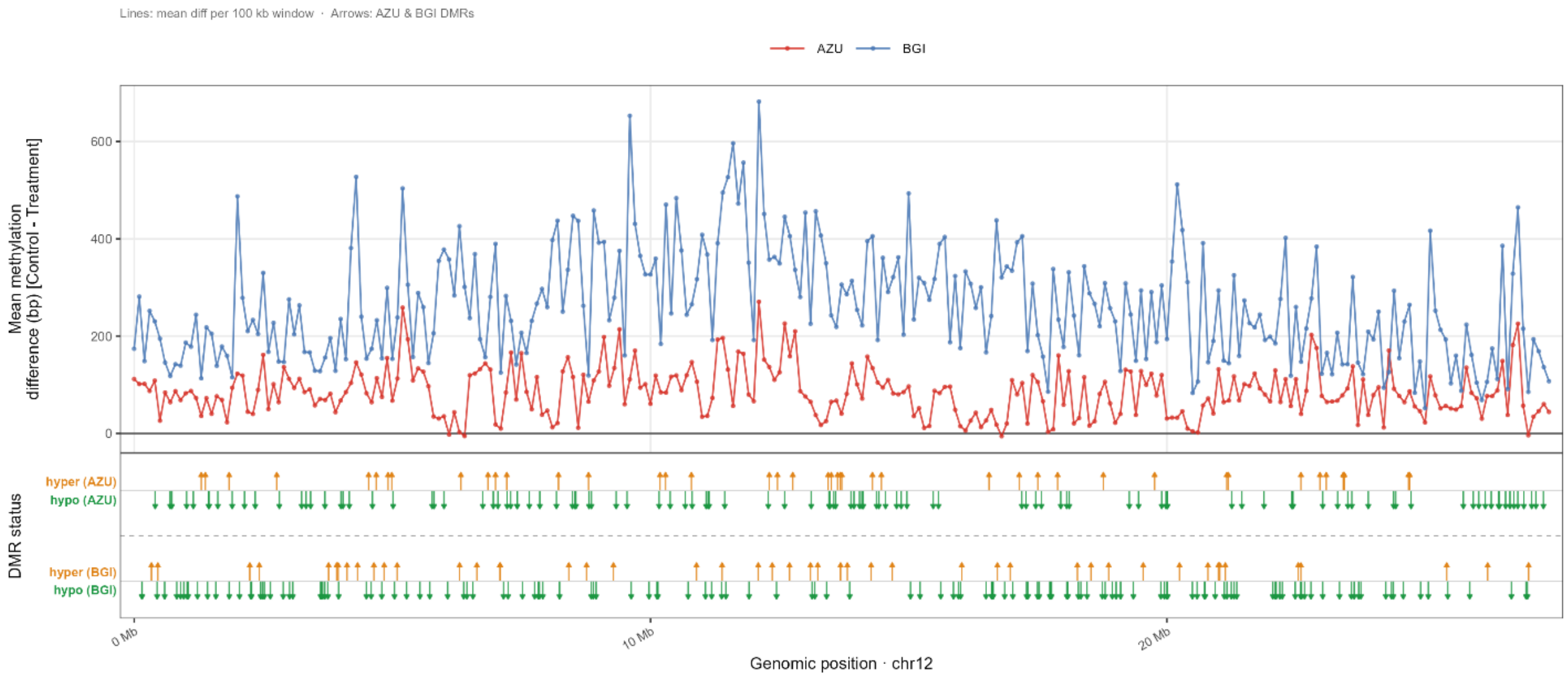
Lines: mean diff per 100 kb window · Arrows: AZU & BGI DMRs
AZU
BGI
Mean methylation difference (bp) [Control - Treatment]
600
400
200
0
DMR status
hyper (AZU)
hypo (AZU)
hyper (BGI)
hypo (BGI)
0 Mb
10 Mb
20 Mb
Genomic position · chr12

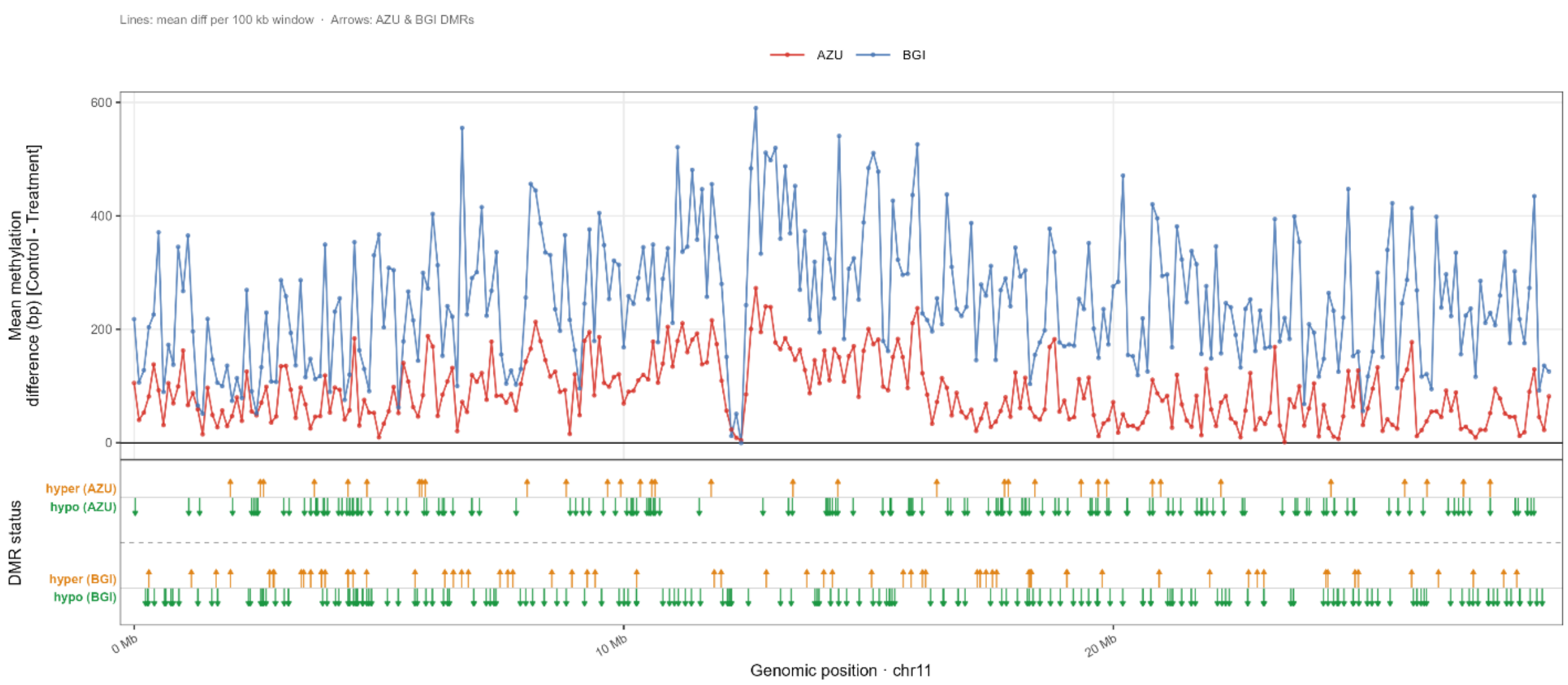
Lines: mean diff per 100 kb window · Arrows: AZU & BGI DMRs
AZU
BGI
Mean methylation difference (bp) [Control - Treatment]
600
400
200
0
DMR status
hyper (AZU)
hypo (AZU)
hyper (BGI)
hypo (BGI)
0 Mb
10 Mb
20 Mb
Genomic position · chr11

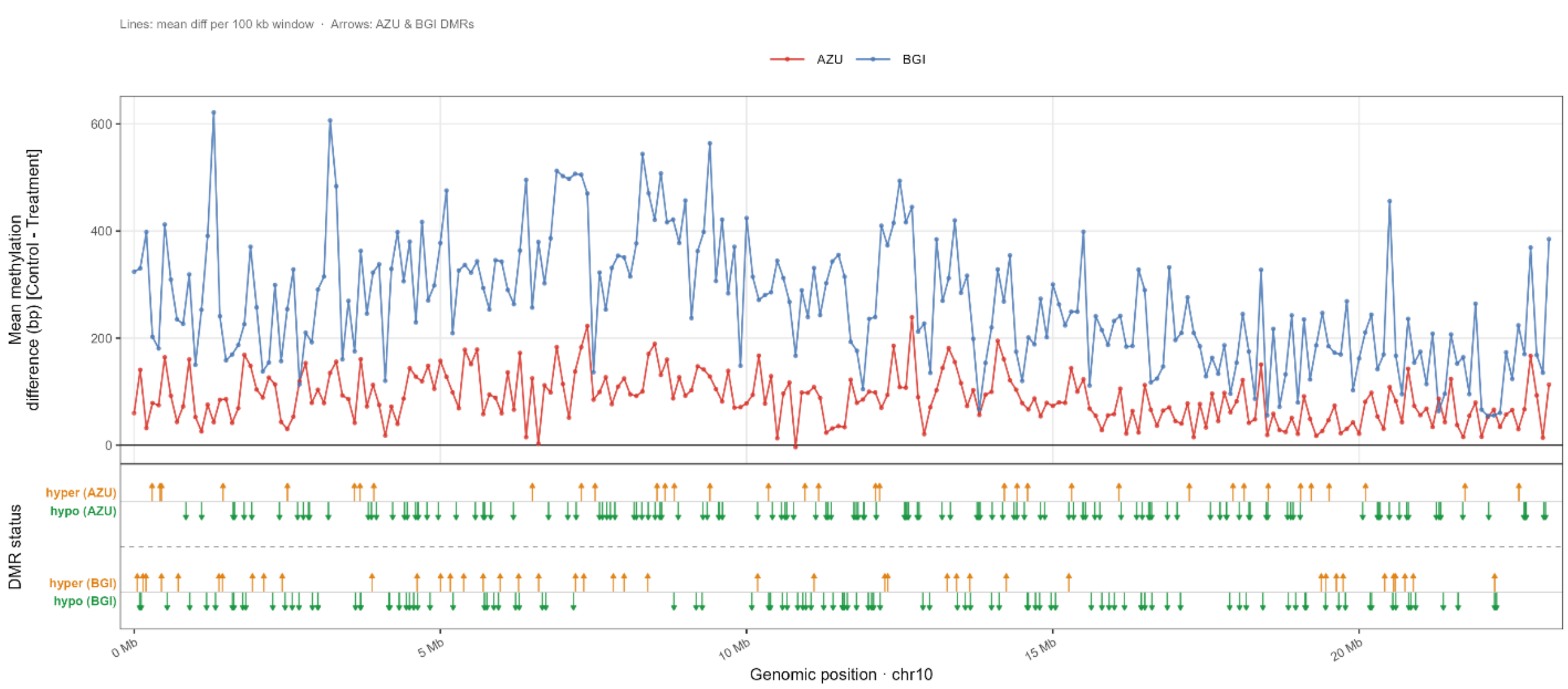
Lines: mean diff per 100 kb window · Arrows: AZU & BGI DMRs
AZU
BGI
Mean methylation difference (bp) [Control - Treatment]
600
400
200
0
DMR status
hyper (AZU)
hypo (AZU)
hyper (BGI)
hypo (BGI)
0 Mb
5 Mb
10 Mb
15 Mb
20 Mb
Genomic position · chr10

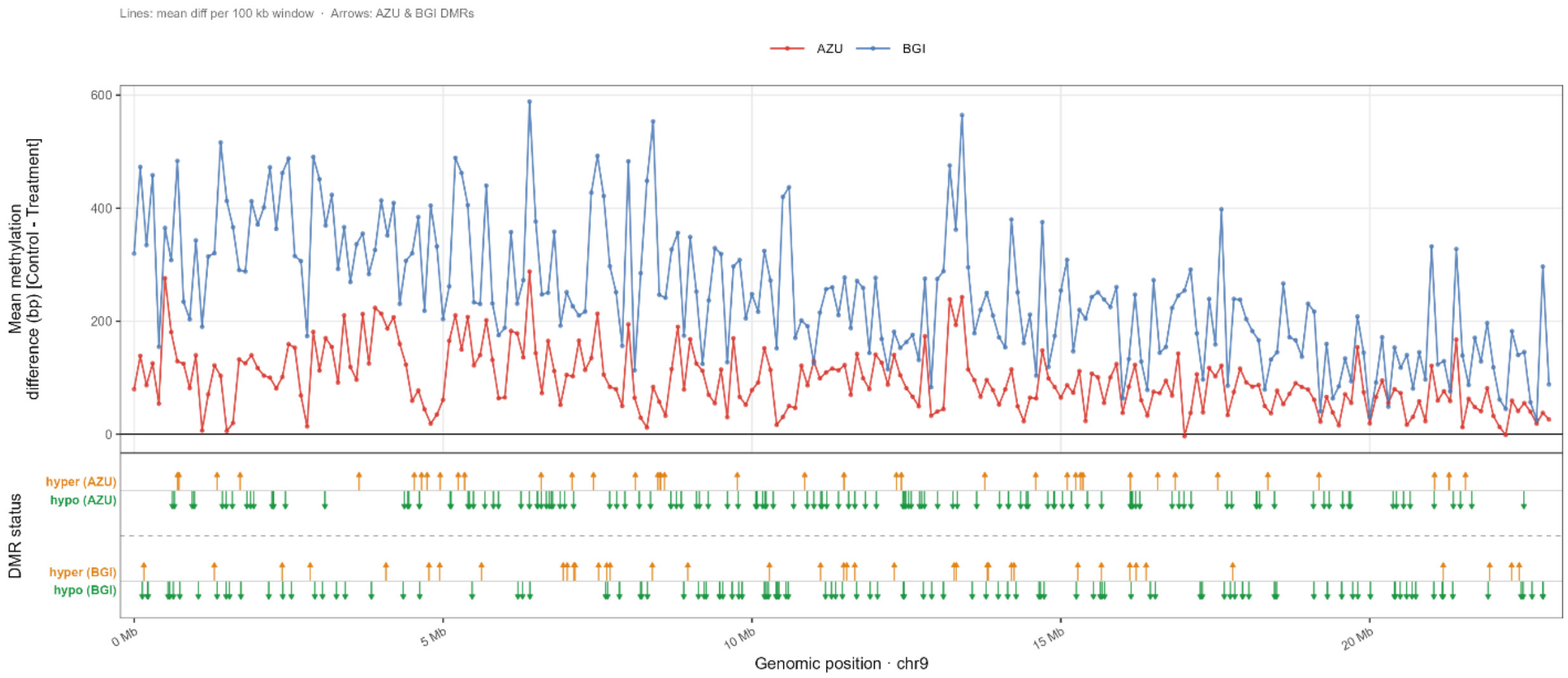
Lines: mean diff per 100 kb window · Arrows: AZU & BGI DMRs
AZU
BGI
Mean methylation difference (bp) [Control - Treatment]
0
200
400
600
DMR status
hyper (AZU)
hypo (AZU)
hyper (BGI)
hypo (BGI)
0 Mb
5 Mb
10 Mb
15 Mb
20 Mb
Genomic position · chr9

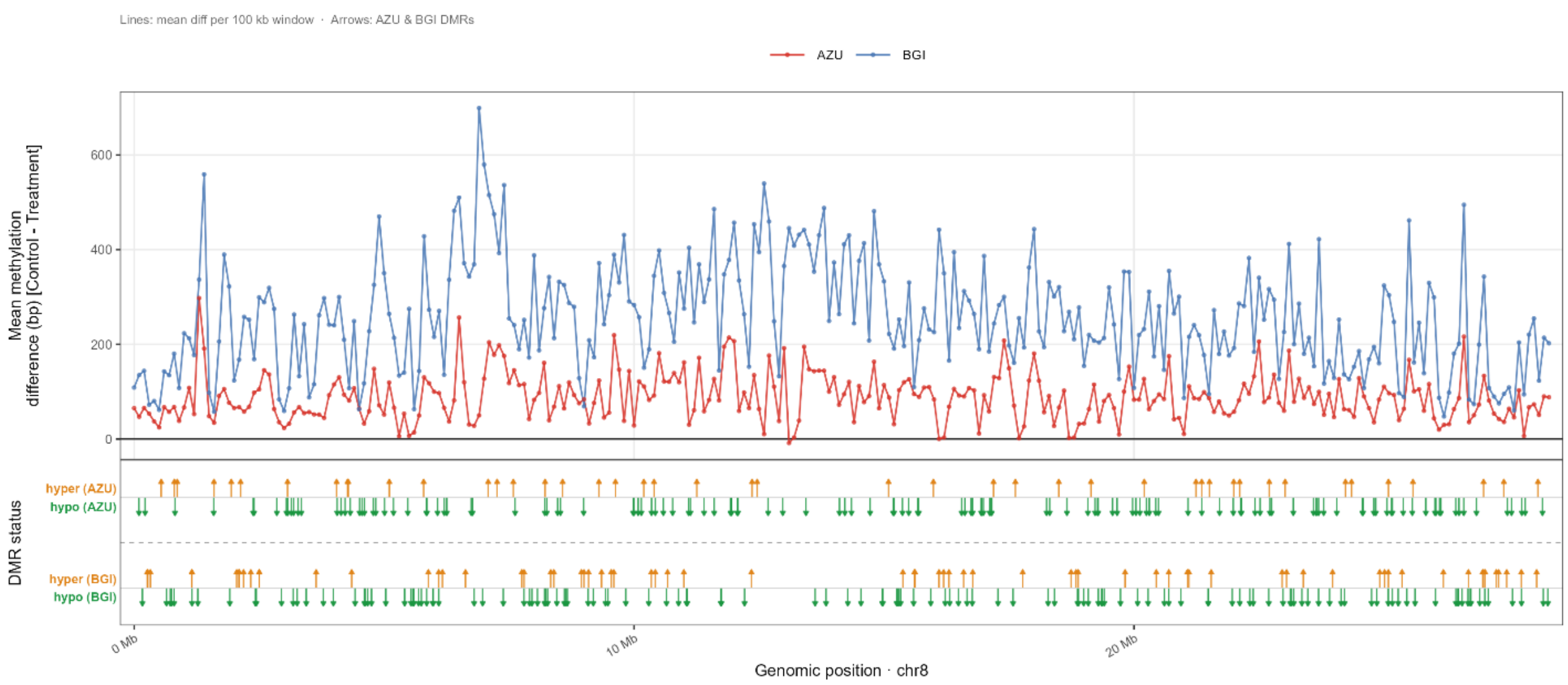
Lines: mean diff per 100 kb window · Arrows: AZU & BGI DMRs
AZU
BGI
Mean methylation difference (bp) [Control - Treatment]
0
200
400
600
DMR status
hyper (AZU)
hypo (AZU)
hyper (BGI)
hypo (BGI)
0 Mb
10 Mb
20 Mb
Genomic position · chr8

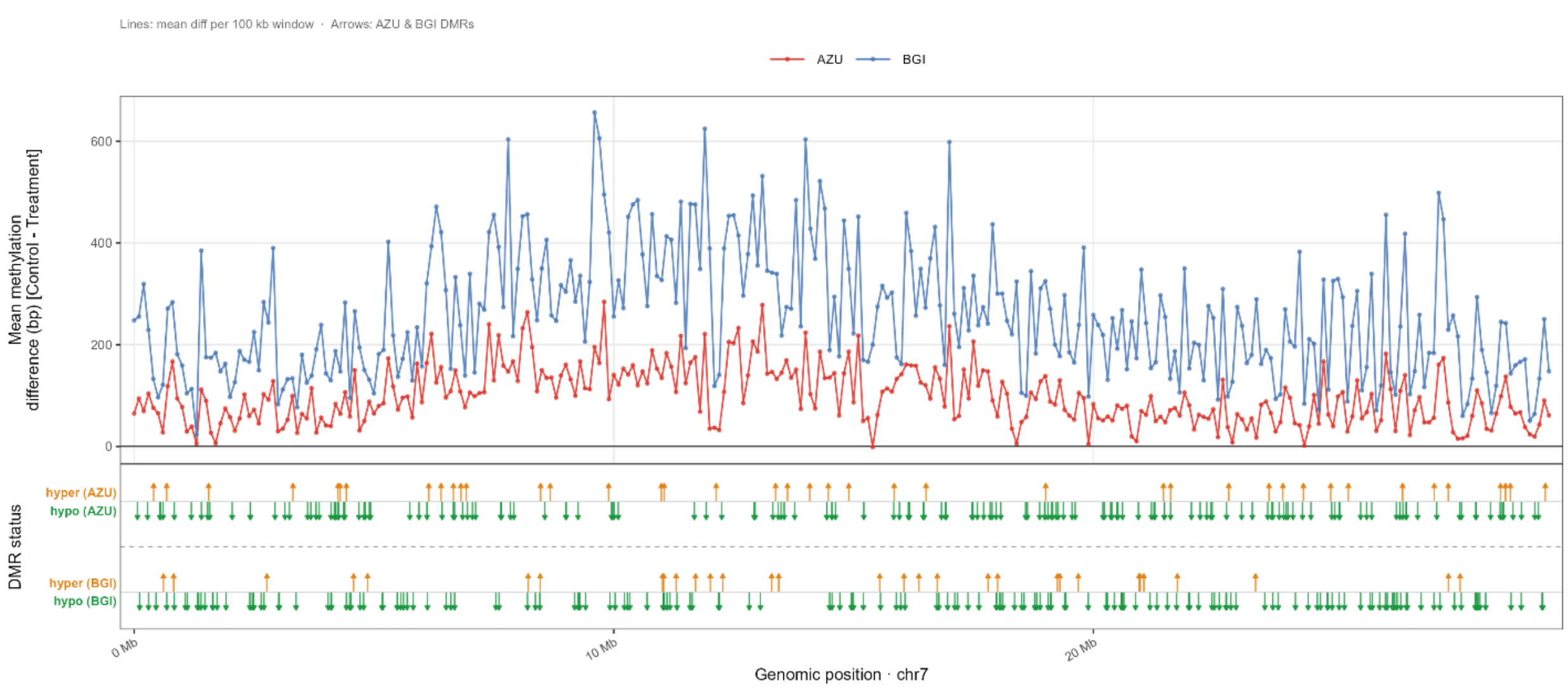
Lines: mean diff per 100 kb window · Arrows: AZU & BGI DMRs
AZU
BGI
Mean methylation difference (bp) [Control - Treatment]
0
200
400
600
DMR status
hyper (AZU)
hypo (AZU)
hyper (BGI)
hypo (BGI)
0 Mb
10 Mb
20 Mb
Genomic position · chr7

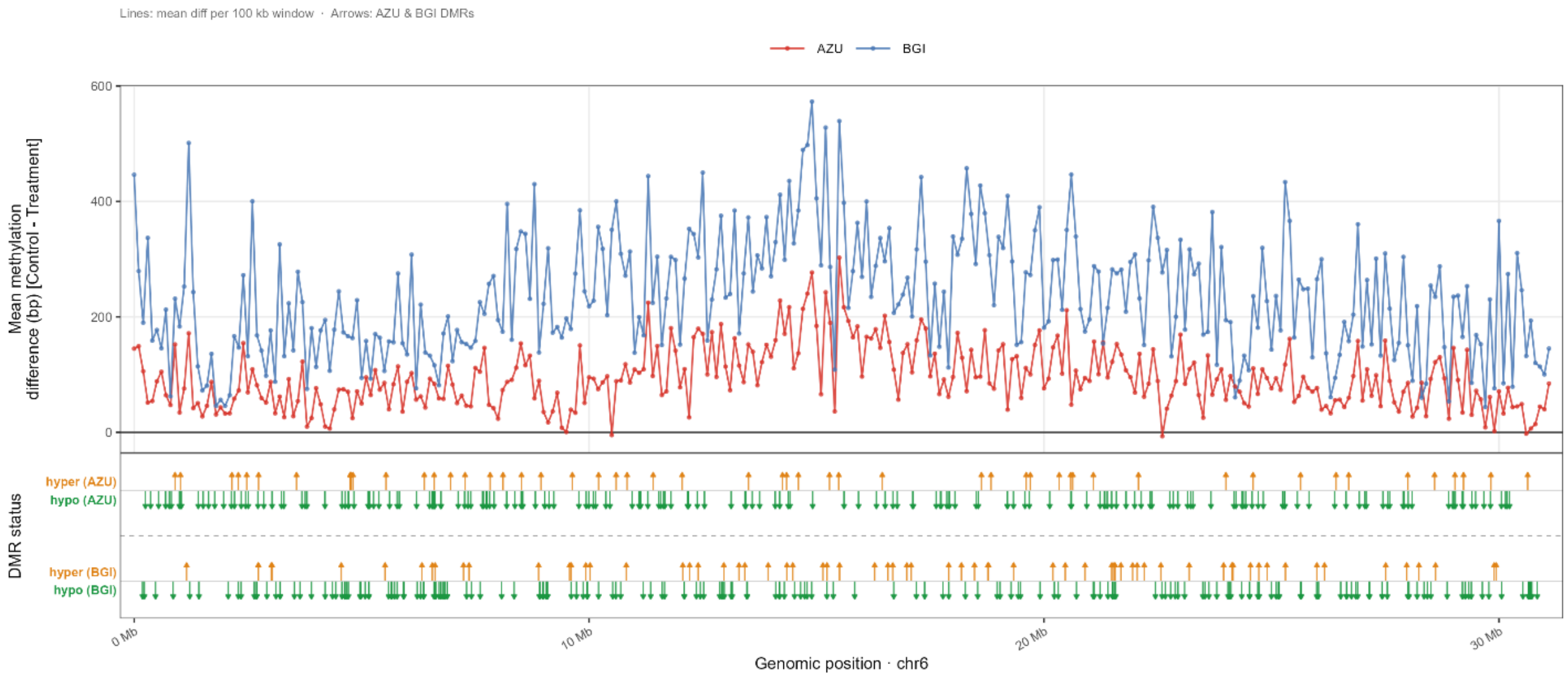
Lines: mean diff per 100 kb window · Arrows: AZU & BGI DMRs
AZU
BGI
Mean methylation difference (bp) [Control - Treatment]
600
400
200
0
DMR status
hyper (AZU)
hypo (AZU)
hyper (BGI)
hypo (BGI)
0 Mb
10 Mb
20 Mb
30 Mb
Genomic position · chr6

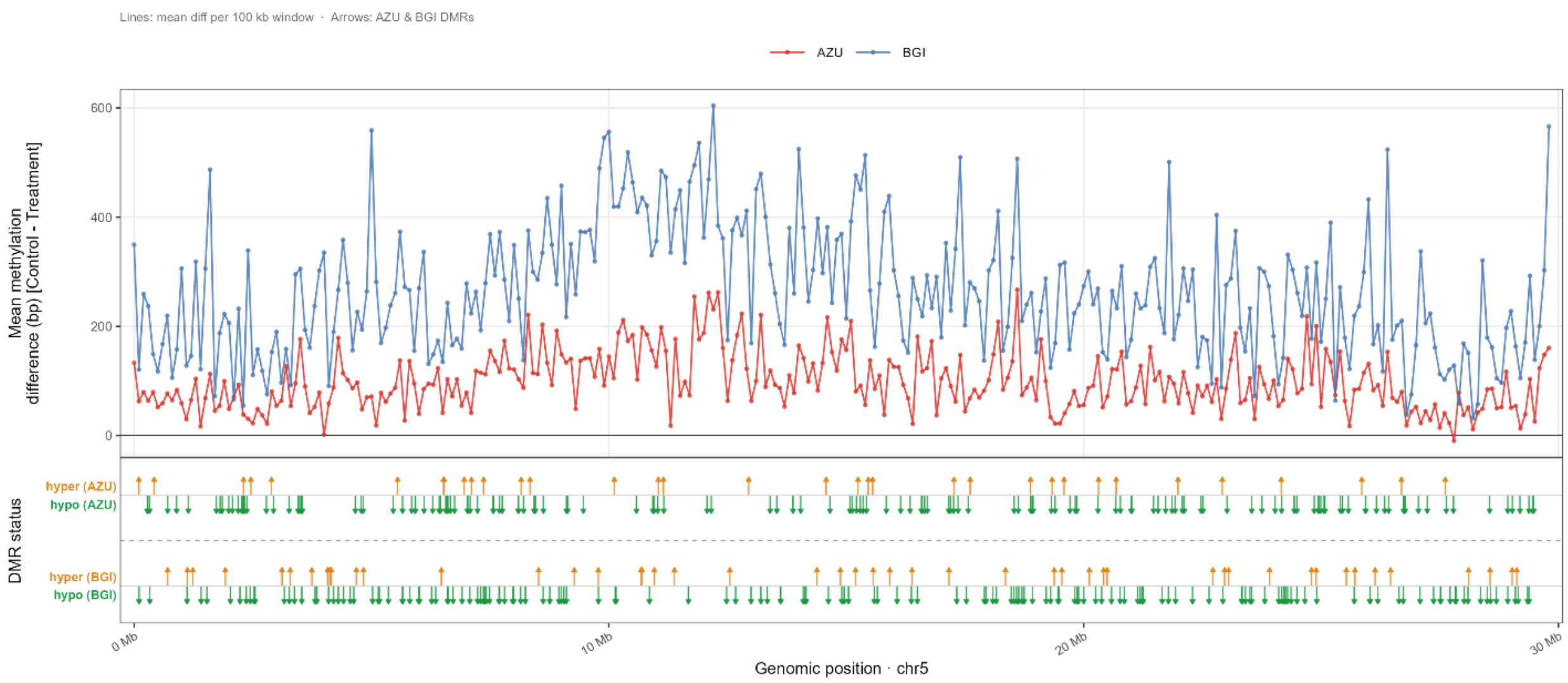
Lines: mean diff per 100 kb window · Arrows: AZU & BGI DMRs
AZU
BGI
Mean methylation difference (bp) [Control - Treatment]
600
400
200
0
DMR status
hyper (AZU)
hypo (AZU)
hyper (BGI)
hypo (BGI)
0 Mb
10 Mb
20 Mb
30 Mb
Genomic position · chr5

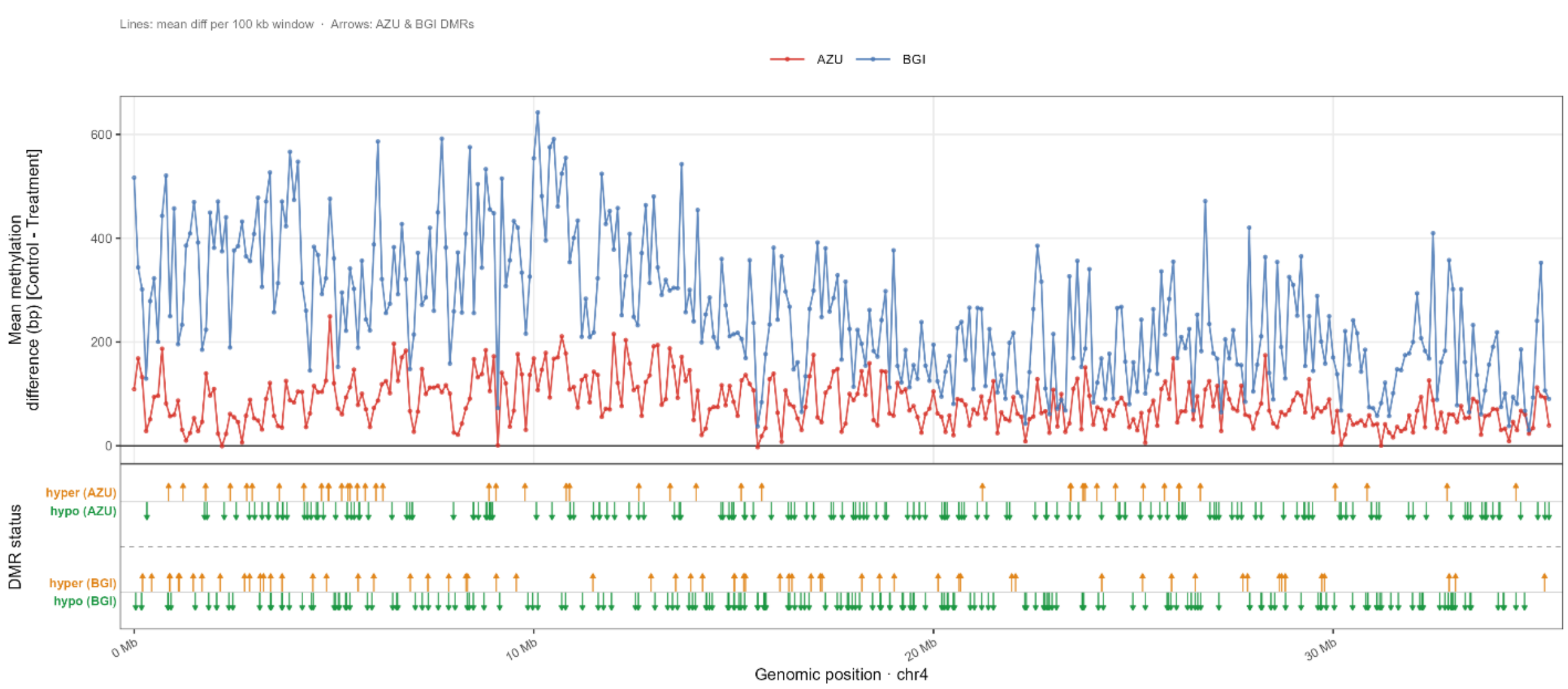
Lines: mean diff per 100 kb window · Arrows: AZU & BGI DMRs
AZU
BGI
Mean methylation difference (bp) [Control - Treatment]
600
400
200
0
DMR status
hyper (AZU)
hypo (AZU)
hyper (BGI)
hypo (BGI)
0 Mb
10 Mb
20 Mb
30 Mb
Genomic position · chr4

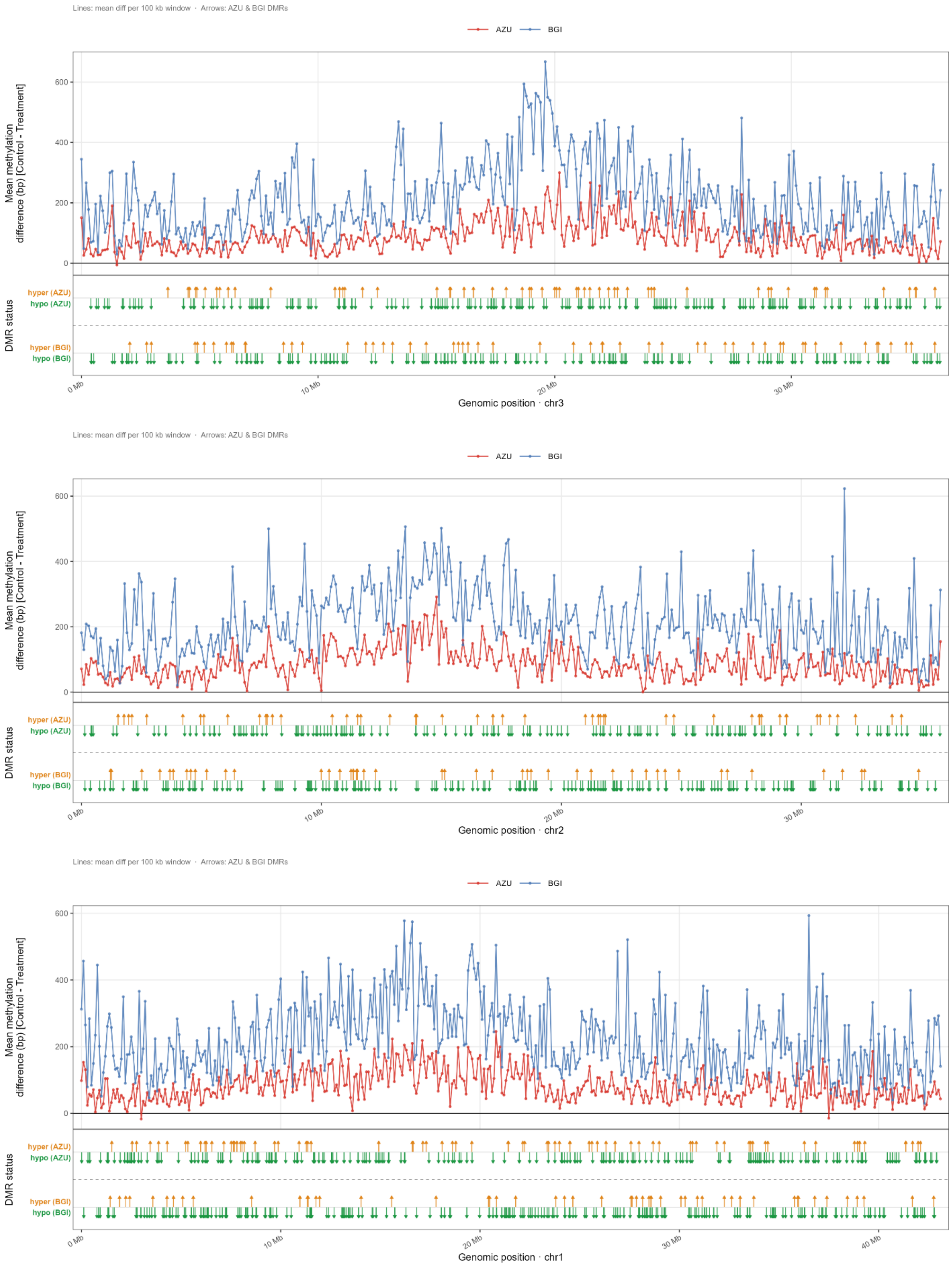

Supplementary Figure S4. Differential CHG methylation and DMR distribution across all 12 chromosomes in AZU and BGI rice genotypes under aluminum stress
