# Supplementary material for: Integrative methylome and transcriptome analysis reveals genotype and sequence context-specific responses to aluminum stress in rice
Source: Front Plant Sci. 2026 Jul 31;17:1896320. doi: 10.3389/fpls.2026.1896320 (PMC13474991; doi:10.3389/fpls.2026.1896320)
Supplement: Supplementary file 5 [file Image5.pdf]

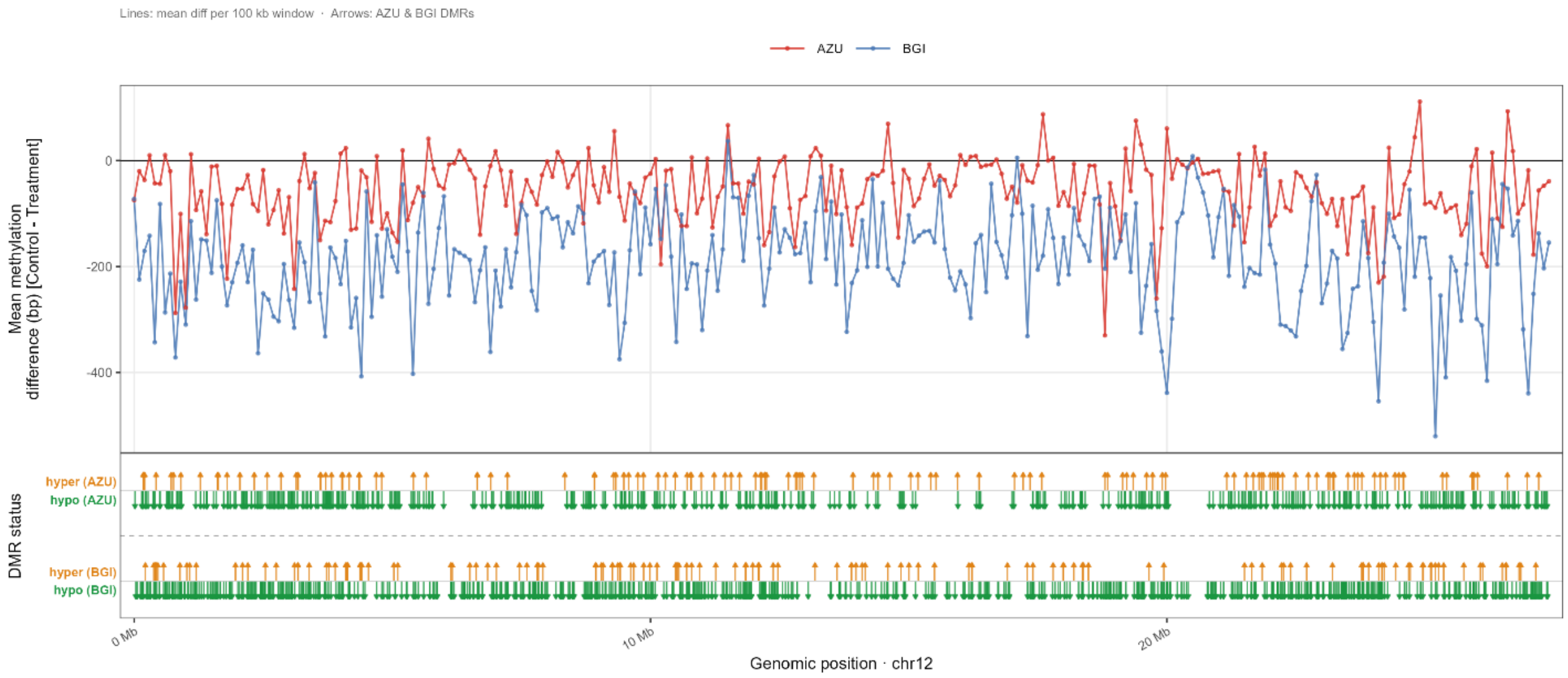
Lines: mean diff per 100 kb window · Arrows: AZU & BGI DMRs
AZU
BGI
Mean methylation difference (bp) [Control - Treatment]
0
-200
-400
DMR status
hyper (AZU)
hypo (AZU)
hyper (BGI)
hypo (BGI)
0 Mb
10 Mb
20 Mb
Genomic position · chr12

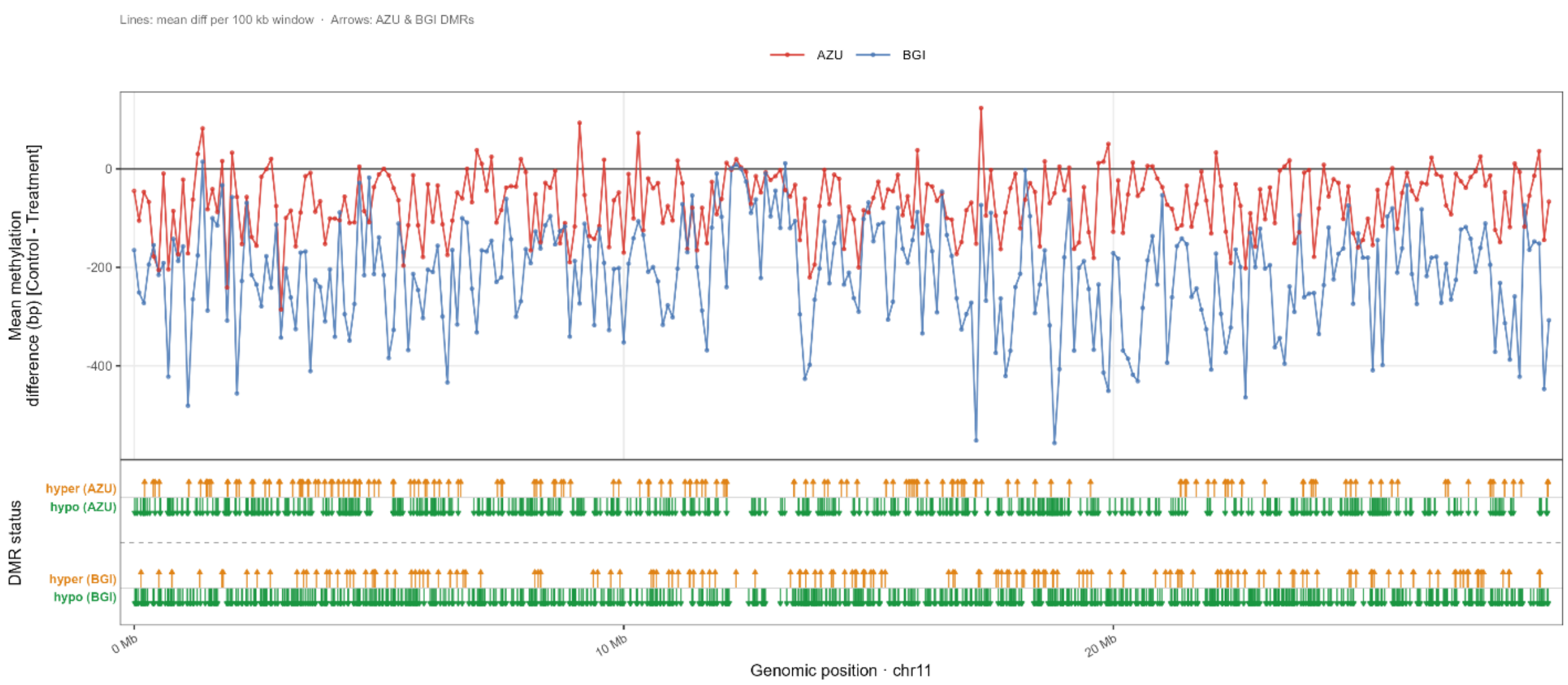
Lines: mean diff per 100 kb window · Arrows: AZU & BGI DMRs
AZU
BGI
Mean methylation difference (bp) [Control - Treatment]
0
-200
-400
DMR status
hyper (AZU)
hypo (AZU)
hyper (BGI)
hypo (BGI)
0 Mb
10 Mb
20 Mb
Genomic position · chr11

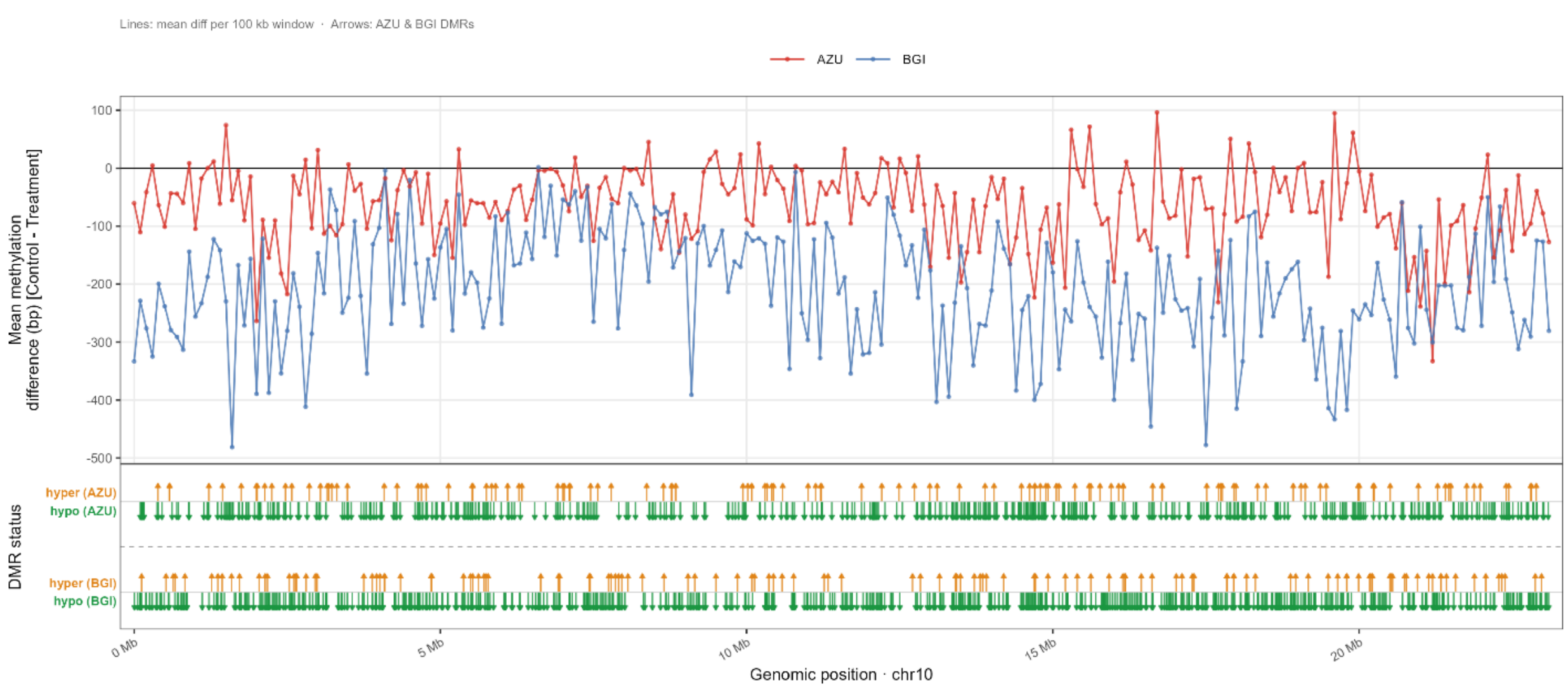
Lines: mean diff per 100 kb window · Arrows: AZU & BGI DMRs
AZU
BGI
Mean methylation difference (bp) [Control - Treatment]
100
0
-100
-200
-300
-400
-500
DMR status
hyper (AZU)
hypo (AZU)
hyper (BGI)
hypo (BGI)
0 Mb
5 Mb
10 Mb
15 Mb
20 Mb
Genomic position · chr10

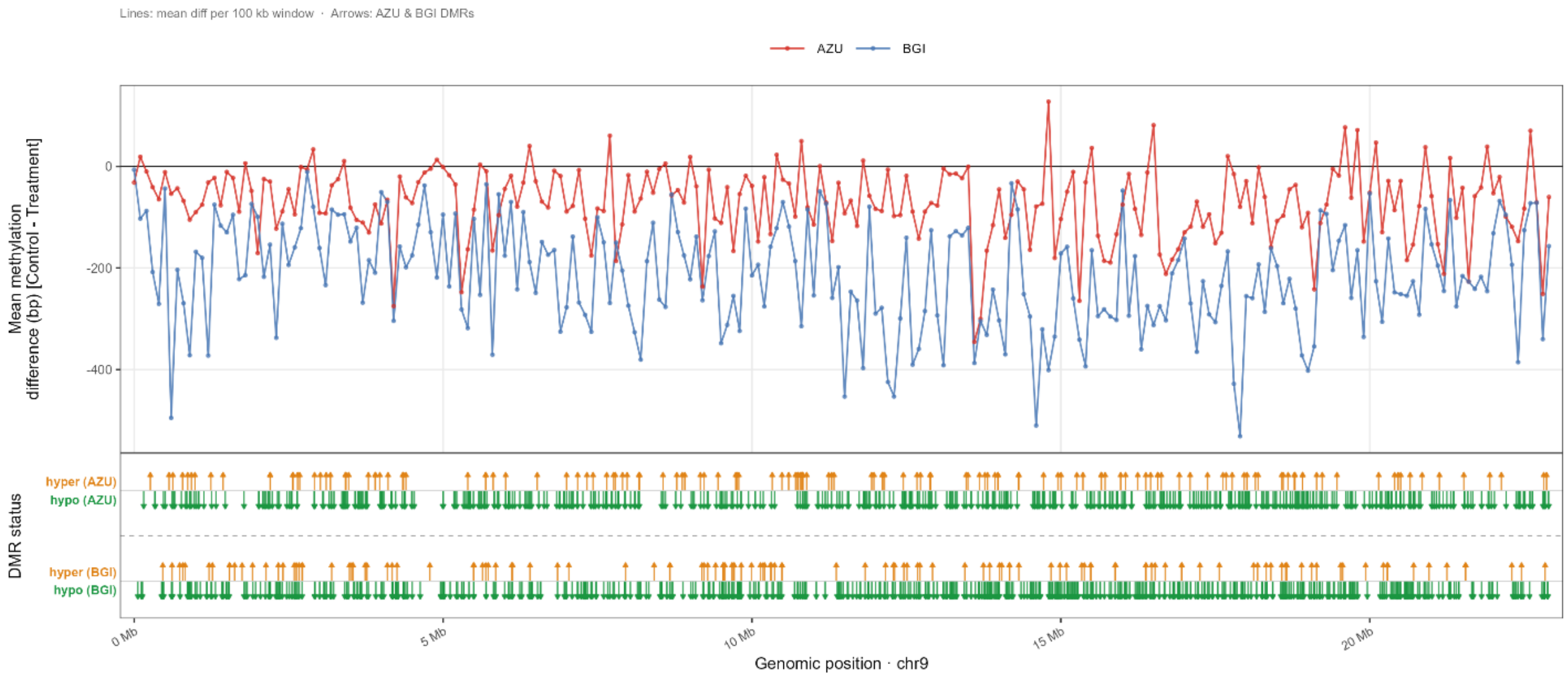
Lines: mean diff per 100 kb window · Arrows: AZU & BGI DMRs
AZU
BGI
Mean methylation difference (bp) [Control - Treatment]
0
-200
-400
DMR status
hyper (AZU)
hypo (AZU)
hyper (BGI)
hypo (BGI)
0 Mb
5 Mb
10 Mb
15 Mb
20 Mb
Genomic position · chr9

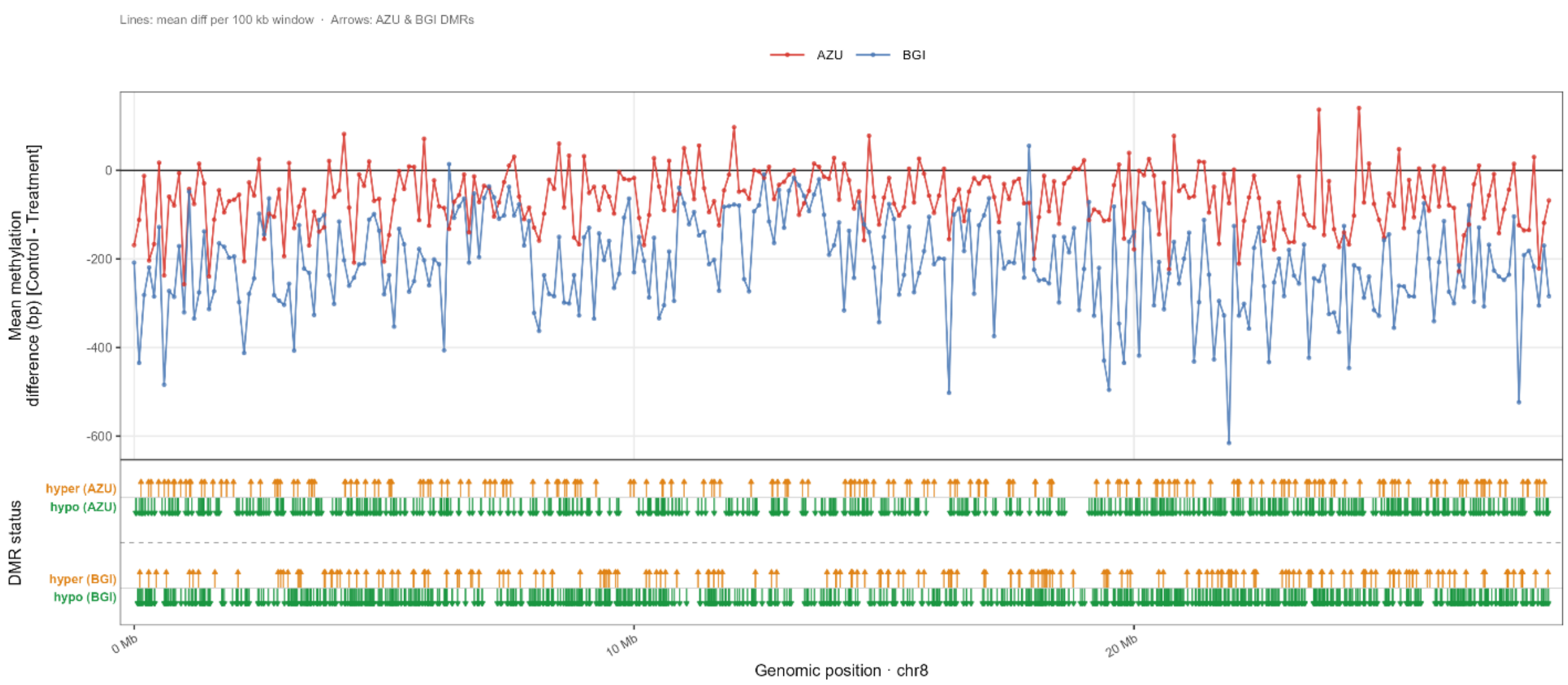
Lines: mean diff per 100 kb window · Arrows: AZU & BGI DMRs
AZU
BGI
Mean methylation difference (bp) [Control - Treatment]
0
-200
-400
-600
DMR status
hyper (AZU)
hypo (AZU)
hyper (BGI)
hypo (BGI)
0 Mb
10 Mb
20 Mb
Genomic position · chr8

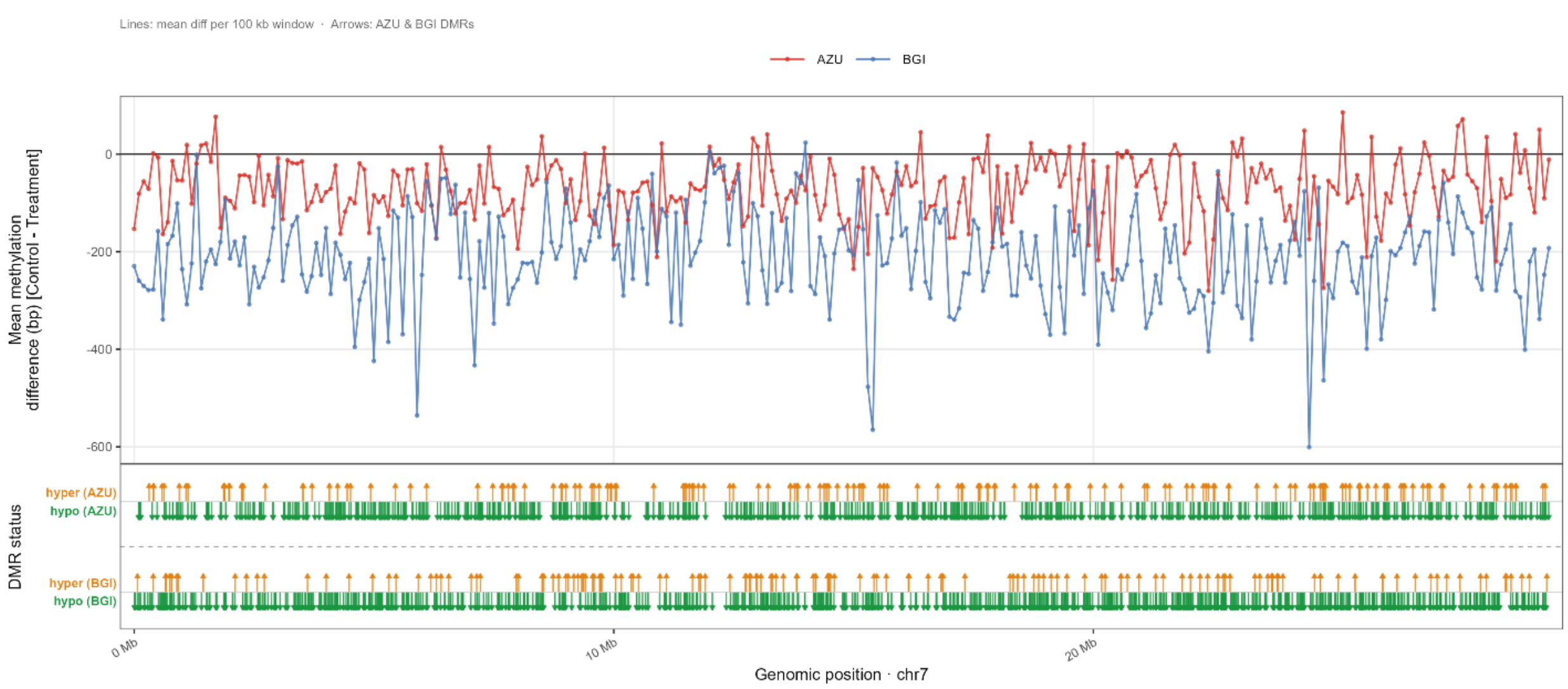
Lines: mean diff per 100 kb window · Arrows: AZU & BGI DMRs
AZU
BGI
Mean methylation difference (bp) [Control - Treatment]
0
-200
-400
-600
DMR status
hyper (AZU)
hypo (AZU)
hyper (BGI)
hypo (BGI)
0 Mb
10 Mb
20 Mb
Genomic position · chr7

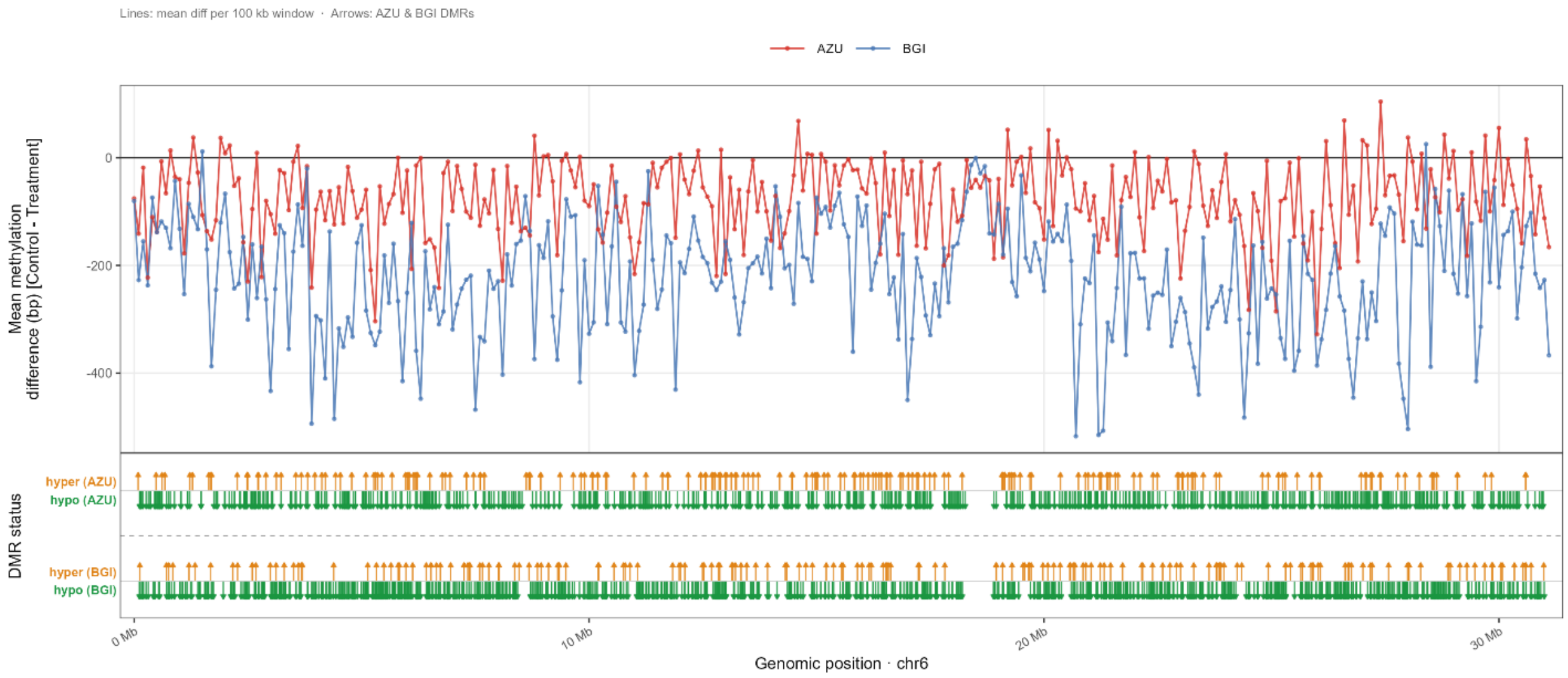

Lines: mean diff per 100 kb window · Arrows: AZU & BGI DMRs
AZU
BGI
Mean methylation difference (bp) [Control - Treatment]
0
-200
-400
DMR status
hyper (AZU)
hypo (AZU)
hyper (BGI)
hypo (BGI)
0 Mb
10 Mb
20 Mb
30 Mb
Genomic position · chr6

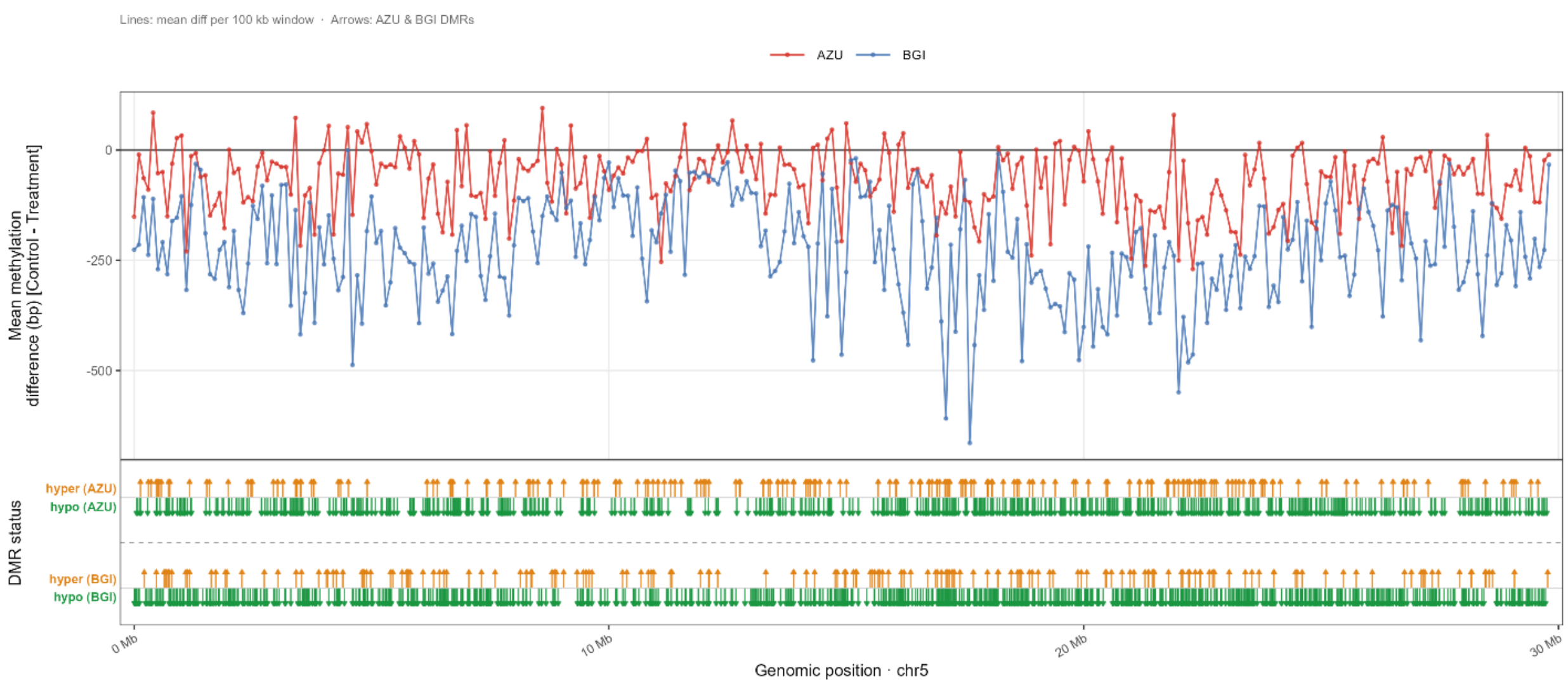

Lines: mean diff per 100 kb window · Arrows: AZU & BGI DMRs
AZU
BGI
Mean methylation difference (bp) [Control - Treatment]
0
-250
-500
DMR status
hyper (AZU)
hypo (AZU)
hyper (BGI)
hypo (BGI)
0 Mb
10 Mb
20 Mb
30 Mb
Genomic position · chr5

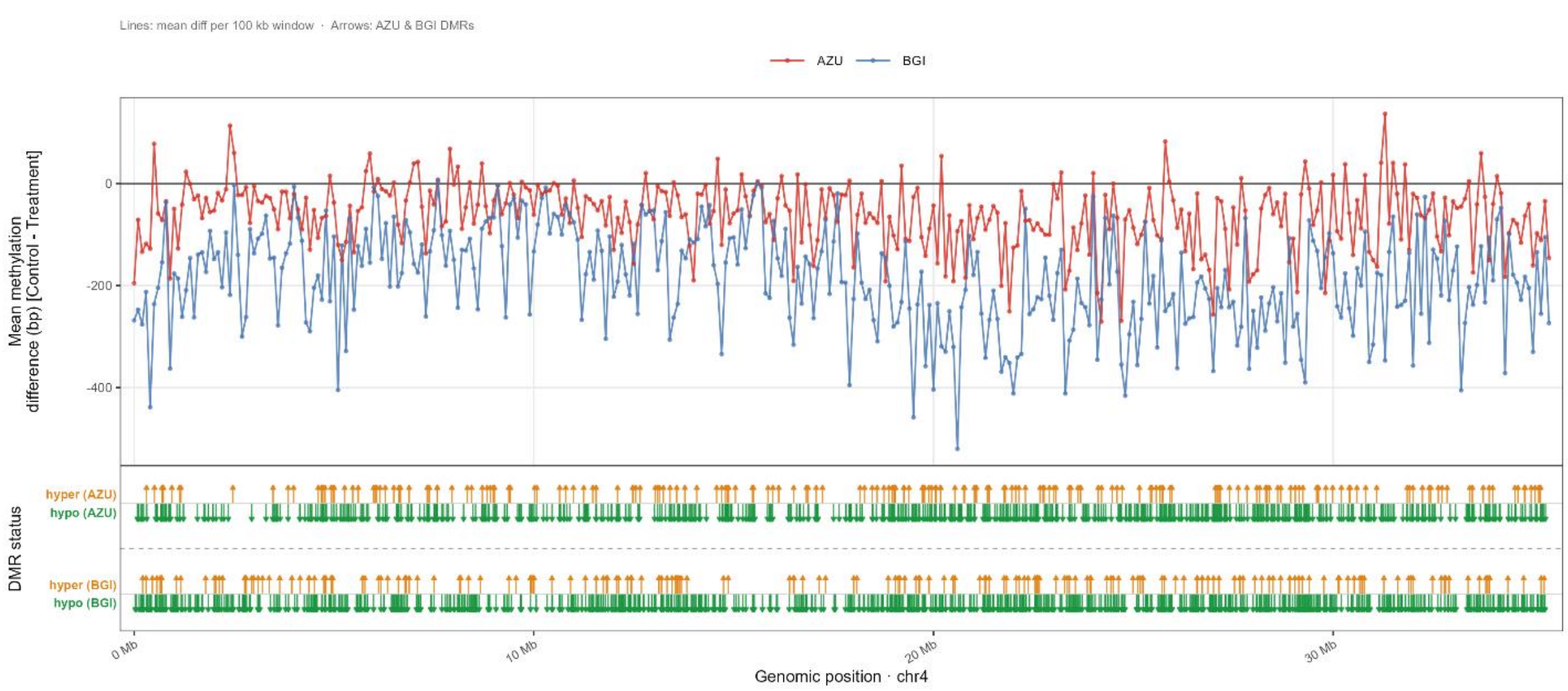

Lines: mean diff per 100 kb window · Arrows: AZU & BGI DMRs
AZU
BGI
Mean methylation difference (bp) [Control - Treatment]
0
-200
-400
DMR status
hyper (AZU)
hypo (AZU)
hyper (BGI)
hypo (BGI)
0 Mb
10 Mb
20 Mb
30 Mb
Genomic position · chr4

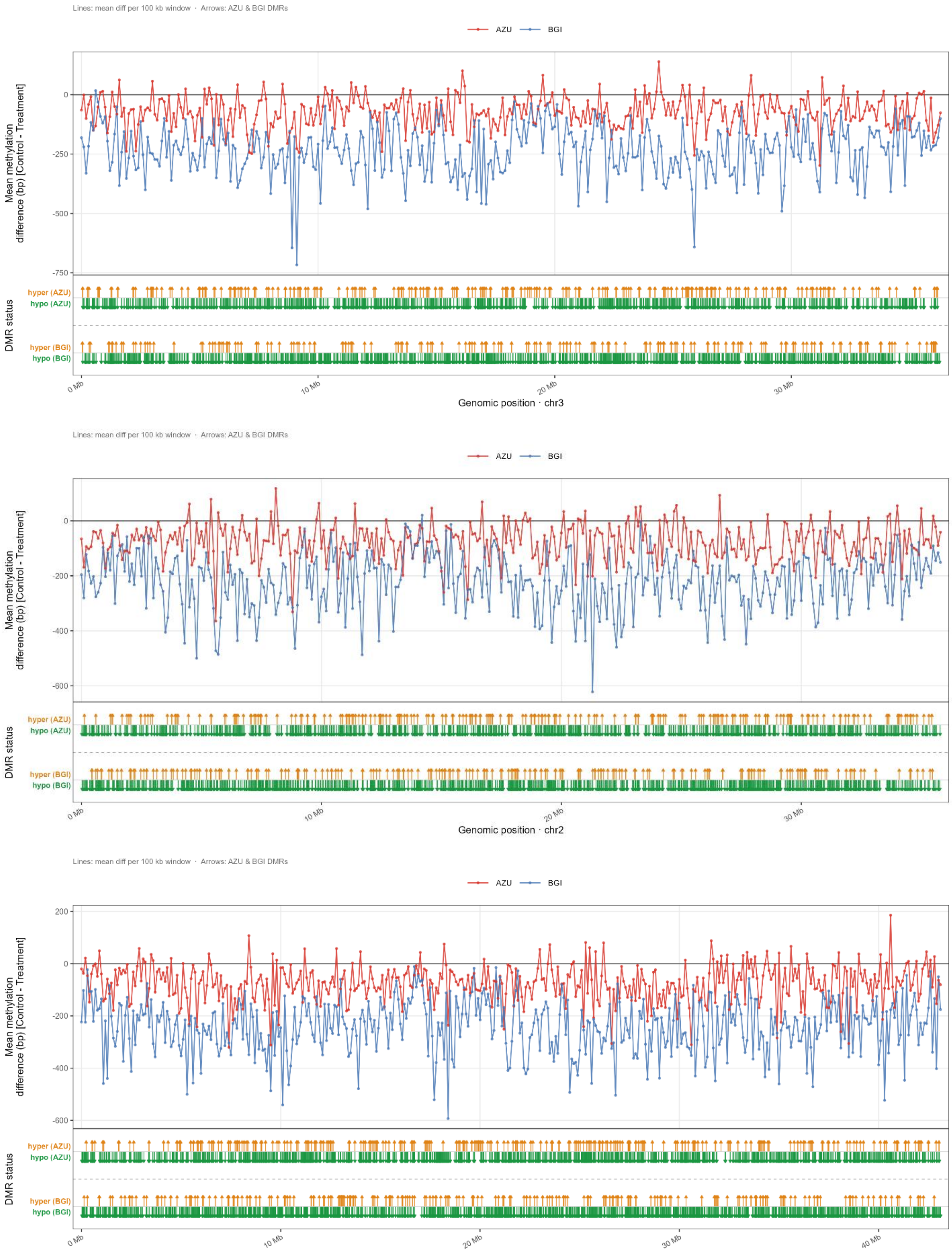

Supplementary Figure S5. Differential CHH methylation and DMR distribution across all 12 chromosomes in AZU and BGI rice genotypes under aluminum stress
